# Mechanical force drives the initial mesenchymal-epithelial interaction during skin organoid development

**DOI:** 10.7150/thno.83217

**Published:** 2023-05-11

**Authors:** Mengyue Wang, Xun Zhou, Siyi Zhou, Miaomiao Wang, Jingwei Jiang, Wang Wu, Tiantian Liu, Wei Xu, Jinwei Zhang, Deming Liu, Yi Zou, Weiming Qiu, Man Zhang, Weiwei Liu, Zeming Li, Dehuan Wang, Tingting Li, Ji Li, Wanqian Liu, Li Yang, Mingxing Lei

**Affiliations:** 1 111 Project Laboratory of Biomechanics and Tissue Repair & Key Laboratory of Biorheological Science and Technology of Ministry of Education, College of Bioengineering, Chongqing University, Chongqing 400044, China.; 2 Department of Dermatology and Cosmetology, Chongqing Hospital of Traditional Chinese Medicine, Chongqing 400021, China.; 3 Three Gorges Hospital, Chongqing University, Chongqing 404000, China.; 4 Department of Burns and Plastic Surgery, Wuhan General Hospital of Chinese People's Liberation Army, Wuhan 430000, China.; 5 Hunan Key Laboratory of Aging Biology, Xiangya Hospital, Central South University, Changsha 410008, China.

**Keywords:** Skin cyst, Dermal cell attachment, Calcium, Piezo1, Mechanical stretch

## Abstract

**Rationale:** Stem cells self-organize to form organoids that generate mini-organs that resemble the physiologically-developed ones. The mechanism by which the stem cells acquire the initial potential for generating mini-organs remains elusive. Here we used skin organoids as an example to study how mechanical force drives initial epidermal-dermal interaction which potentiates skin organoids to regenerate hair follicles.

**Methods:** Live imaging analysis, single-cell RNA-sequencing analysis, and immunofluorescence were used to analyze the contractile force of dermal cells in skin organoids. Bulk RNA-sequencing analysis, calcium probe detection, and functional perturbations were used to verify that calcium signaling pathways respond to the contractile force of dermal cells. *In vitro* mechanical loading experiment was used to prove that the stretching force triggers the epidermal Piezo1 expression which negatively regulates dermal cell attachment. Transplantation assay was used to test the regenerative ability of skin organoids.

**Results:** We found that dermal cell-derived contraction force drives the movement of dermal cells surrounding the epidermal aggregates to trigger initial mesenchymal-epithelial interaction (MEI). In response to dermal cell contraction force, the arrangement of the dermal cytoskeleton was negatively regulated by the calcium signaling pathway which further influences dermal-epidermal attachment. The native contraction force generated from the dermal cell movement exerts a stretching force on the adjacent epidermal cells, activating the stretching force sensor Piezo1 in the epidermal basal cells during organoid culture. Epidermal Piezo1 in turn drives strong MEI to negatively regulate dermal cell attachment. Proper initial MEI by mechanical-chemical coupling during organoid culture is required for hair regeneration upon transplantation of the skin organoids into the back of the nude mice.

**Conclusion:** Our study demonstrated that mechanical-chemical cascade drives the initial event of MEI during skin organoid development, which is fundamental to the organoid, developmental, and regenerative biology fields.

## Introduction

Skin appendages such as hair follicles are required for defense, thermoregulation, sensing, metabolism, etc. for mammals. Millions of people worldwide suffer from severe skin injury without skin appendage regeneration during healing. Recent studies have made progress in establishing skin organoids that form skin and hair follicles *in vitro* and *in vivo* 1-4, providing promising therapeutic potential for clinical application to treat skin injury and hair loss. Skin organoids can be generated by 3-dimensional (3D) culture of the induced pluripotent stem cells (iPSC), embryonic stem cells, or skin progenitor cells 1-4. We have established a skin organoid culture system, in which dissociated epidermal and dermal cells isolated from newborn mouse skin are remixed and cultured in a transwell insert 1, 5. The dissociated cells undergo prominent consecutive morphological phase transitions including dissociation, aggregation, polarization, coalescence, planarization, and hair primordia formation. Transplantation of the skin organoids into the back of nude mice results in robust hair regeneration 1.

Epithelial organoid development resembles embryonic development, in which mesenchymal-epithelial interactions (MEIs) trigger the various organs formation through specific molecular events 6. MEI governs the bud development of teeth 7 and lacrimal glands 8, duct morphogenesis of the lung 9 and kidney 10 and stomach specification and regionalization 11. MEI is also required for hair follicle morphogenesis 6. Since the skin organoid culture system is mainly composed of epidermal and dermal cells, thus in the present study, we asked whether and how MEI initiates the competency of skin progenitor cells during organoid culture, which influences prospective hair follicle development after graft.

Mechanical force is critical for embryonic development, tissue regeneration, and organoid formation 12-15. During pre-implantation development, the self-organization of the embryo into the blastocyst relies on intrinsic contractile force generated by the asymmetric splitting of the apical domain 16. Mechanical stretch of mouse dorsal skin stimulates the production of chemokines which activate macrophages to release growth factors that induce hair regeneration 17. The mechanical force has been shown to control stem cell expansion during intestinal organoid formation 18. At the molecular level, mechanosensitive molecules such as integrins 19, cadherin complexes 20-22, and ion channels 23, 24, can respond to mechanical force to affect cell behavior and tissue regeneration. For instance, intracellular calcium and cytoskeleton are involved in mechanical signaling transduction 25. Mechanical traction or shear stress can rapidly increase intracellular calcium (Ca2+) concentration in osteoblasts and force-sensitive cells (such as vascular endothelial cells). Mechanical stress induces the Ca2+-dependent signaling pathway in erythroblasts and promotes erythropoiesis 26.

Piezo1 has been identified as a mechano-active cation channel factor. Recent studies have shown that mechanically activated piezo channels modulate outflow tract valve development and aortic valves 27-29. Mechanical stimulates Piezo1-mediated Ca2+ influx which facilitates sprouting angiogenesis 30. Mechanical signaling via Piezo1 keeps muscle stem cells in a quiescent state and prevents senescence 31. Neural stem cells respond to cell membrane tension through a stretch-activated ion channel Piezo1 32. These studies suggest that Piezo1 is largely involved in mechanobiology.

The skin organoids can secrete extracellular matrix (ECM) which polymerizes and matures during culture 1. It is reasonable to speculate that the tissue mechanical environment is changing spatiotemporally. We hypothesized that the ever-changing ECM environment influences the self-organization of skin organoids, and potentiates them to form hair follicles through MEI 1. In the present study, we observed that the dermal cells first attach to the epidermal aggregate in day 1 (D1) skin organoid culture, indicating the initial MEI event during organoid culture. Eventually, more dermal cells are attached to the epidermal cyst, forming a skin sphere that includes an epidermal cyst, several layers of surrounding dermal cells, and a basement membrane lying between at D3, suggesting a strong MEI which generates the regenerative potential. Therefore, this study aims to investigate the mechanism by which dermal cells-derived mechanical force drives MEI which permits self-organization in skin organoid culture and hair follicle formation upon transplantation.

## Materials and methods

### Mice

CD1, K14H2BGFP, and nude mice were used in this study. CD1 and nude mice were purchased from Laibite Biotechnology company (Chongqing, China). K14H2BGFP mice were kindly gifted by Dr. Ting Chen at the National Institute of Biological Sciences (Beijing, China). Mice were kept at 25°C with a 12 h light cycle, with food and water supply. All performed procedures were approved by the Institutional Animal Care and Use Committees of Chongqing University.

### Skin organoid culture

The method for primary newborn mouse cell culture can be referred to our previous publication 1. Briefly, cells were derived from the dorsal skin of newborn mice within 24 h after birth, and the dermis and epidermis were separated by floating the dorsal skin overnight in 0.25% trypsin (#15050057, Gibco, USA) solution at 4°C. Epidermal cells are obtained by scissors cutting, filtering, and centrifuging. The dermal cells were digested in 0.35% collagenase I (#LS004197, Worthington, USA) for 20 min, and then obtained by filtration and centrifugation. The dissociated epidermal cells and dermal cells were mixed at a ratio of 1:9 and were dropped onto the upper chamber of a Transwell culture, and the lower chamber was filled with 700ul DMEM/F12 (#MT10013CV, Corning, USA) culture medium containing 10% FBS (#10099-141C, Gibco, USA). The cells were cultured in an incubator with 5% CO2 at 37 °C, with the culture medium being changed every other day.

### Real-time quantitative reverse transcription PCR (RT-qPCR)

After being subjected to stretching for 12 h, the total RNA of the skin organoid culture was harvested by Trizol reagent (#NR0002, Leagene, China) according to manufacturers' instructions. The RNA was then converted to cDNA using a ReverTra Ace RT-qPCR kit (#RR047Q, Takara, Japan) according to the manufacturer's protocol. Gene expression was analyzed by RT-qPCR using the SYBR Green PCR Master Mix (#RR820A, Takara, Japan); Gapdh was used as an internal control. The primers were shown in Table S1. The gene expression levels for each amplification were calculated using the ΔΔCT method and normalized against Gapdh mRNA and all reactions were run in triplicate.

### Immunofluorescence staining

Cell cultures were fixed in 4% (v/v) paraformaldehyde (PFA, #P804537, Macklin, China) at 4°C overnight. Paraffin-embedded tissue was cut into 6 um sections. The samples were dewaxed and rehydrated, and antigen was retrieved with citric acid (#C805019, Macklin, China) and sodium citrate (#S818273, Macklin, China) solution. Then the samples were blocked with 2% bovine serum albumin (BSA; #A8020, Solarbio, China) buffer at 37°C for 1 h, followed by incubation of primary antibodies at 4°C overnight. The primary antibodies were shown in Table S2. The samples were incubated with fluorescently-labeled secondary antibodies (Alexa Fluor 488-conjugated goat anti-mouse IgG (Beyotime, China) or 488-conjugated goat anti-mouse IgG (Beyotime, China) at 1:500 in PBS at 37°C for 2 h. Samples were washed and incubated with propidium iodide (PI) at room temperature for 20 min. Finally, drop glycerol for mounting. Images were taken under a confocal microscope (Leica, Germany) at Chongqing University Analysis and Testing Center.

### Hematoxylin and eosin (H&E) staining

The samples were stained with hematoxylin and eosin at room temperature for 2 min, then soaked in tap water for 5 min, and finally dehydrated, mounted with glycerol. Images were taken under a phase-contrast microscope (Motic, China).

### Wholemount immunofluorescence staining

Wholemount immunofluorescence staining was performed using a previously published method 33. In brief, cell cultures were fixed in 4% PFA buffer at 4°C overnight, permeabilized, descaled, incubated with primary antibodies (Table S2), incubated with fluorescence-labeled secondary antibodies, re-fixed in 4% (v/v) PFA, penetrated with Triton-100, and photographed with a Leica inverted microscope (Leica, Germany) for Z-axis slice scanning. Three-dimensional reconstruction was performed with the Leica-associated software.

### Intracellular calcium concentration measurement with Fluo-4 AM fluorescent probe

Cytosolic calcium signals were measured with Fluo-4 AM (#S1060, Beyotime, China) according to the manufacturer's instructions. In detail, cell cultures were incubated with Fluo-4 AM (final concentration of 4 μM) for 30 min in PBS at 37°C, then washed 3 times with PBS and incubated for an additional 15 min in the absence of Fluo-4AM to complete the de-esterification process of the dye. Images were obtained by acquiring emission at 488 nm under a confocal microscope (Leica, Germany).

### Live-Dead staining

Cell viability assay was measured by a Live and Dead Cell Staining Detection Kit (#MA0361, Meilunbio, China) according to the manufacturer's instructions. In detail, cell cultures were incubated with Calcein AM solution (final concentration of 2 μM) and PI solution (final concentration of 8 μM) for 30 min in PBS at 37°C, then washed 3 times with PBS. Images were obtained by acquiring emission at 488 nm and 552 nm under a confocal microscope (Leica, Germany).

### Small molecules perturbation and recombinant protein treatment

After treatment with small molecules or recombinant proteins which were added into the culture medium at different concentrations, the samples were cut into sections at 6um and stained with Collagen I or other antibodies to visualize phenotypic alterations, such as changes in dermal cell attachment and skin cyst size. Cyst cell numbers were counted using ImageJ software. Names, catalog numbers, and concentrations of small molecules are listed in Table S3.

### Application of cyclic mechanical stretch

For the mechanical stretch studies, the dissociated epidermal cells and dermal cells mix were dropped onto collagen I-coated flexible‐bottom six‐well culture plates (BioFlex culture plates, #BF-3001, Flexcell, USA) at a density of 1X 10^7^ cells per well, and were allowed to adhere over 6 h. After 6 h, cell cultures were dropped in DMEM/F12 culture medium containing 10% FBS. Cell cultured on the flexible bottom of six‐well plates were then subjected to cyclic stretching with 10% strain at a frequency of 1.0 Hz for up to 12 h using an FX 4000 Flexcell Tension System (flexcell, Flexcell International Corporation). During the stretch procedure, cells were maintained in an incubator at 37°C with 5% CO2. Control cells were plated in the same plates but were not subjected to stretch. Four different treatment regimens were assigned: (i) non-stretched controls (Static), (ii) cell cultures treated with stretched (10% Stretch), (iii) cell cultures treated with stretched and added Piezo1 inhibitor (10% Stretch+iPiezo1), (iv) cell cultures treated with stretched and added Piezo1 activator (10% Stretch+Yoda1).

### Transplantation

Nude mice were prepared and draped with betadine solution under anesthesia. The skin was excised for 1cm in diameter on both sides of the dorsal back. The cell cultures were transplanted onto the dorsum of nude mice using the planar forming assay procedure 34. Two weeks after transplantation, the bandages were removed and the regenerated hair follicles were photographed and counted.

### Bulk RNA-seq analysis

The RNA-seq data of newborn mouse organoids (GSE86955) were downloaded from our previous publication 1. For clustering analysis, differentially expressed genes among 6 h and D2 skin organoid culture were determined by limma. False discovery rate < 0.05 and log2 fold change > 1 were used as a threshold to determine significant differences in gene expression. Differential expression gene enrichment and functional annotation analysis were performed by R package cluster Profiler 4.0.

### ScRNA-seq analysis

The scRNA-seq data of human skin organoid (GSE147206) were downloaded from GEO database 3. We reanalyzed the skin organoids (D48) data generated using WA25 hESCs cell line. We used Seurat v.4.1.1 to perform QC, normalization, feature selection, linear and non-linear dimensional reduction, cell clustering, finding cluster biomarkers, and assigning cell type identity to clusters. Clusters “EPI” and “FB” were divided by specified markers: COL17A1, KRT1, TYMS, DPT, COL9A2, and ANGPTL1 (Figure 2A). To analyze the interactions between epidermal cells and dermal cells, we used CellChat 35 v.1.4.0 to predict the number and weights/strength of intracellular communications (Figure 2B) from scRNA-seq data. We also employed CellCall 36 v. 0.0.0.9000 to identify the ligand-receptor interactions between epidermal cells and dermal cells (Figure 2C). We selected the “EPI” and “FB” clusters for further analyses. KEGG enrichment analysis (Figure 2D) was performed by https://www.bioinformatics.com.cn, an online platform for data analysis and visualization. We used clusterProfiler v.4.4.4 for another KEGG enrichment analysis and visualization (Figure S2A).

### Statistical analyses

Data were collected from three independent experiments and expressed as mean ± standard deviation (SD). All significance analyses, including one-way analysis of variance (ANOVA), multiple comparisons, and t-tests were performed using Origin 8.0 software. P values were considered significant at 0.05, 0.01, and 0.001 levels.

## Results

### Attachment of dermal cells to the epidermal cyst potentiates hair regeneration

**Figure 1:**
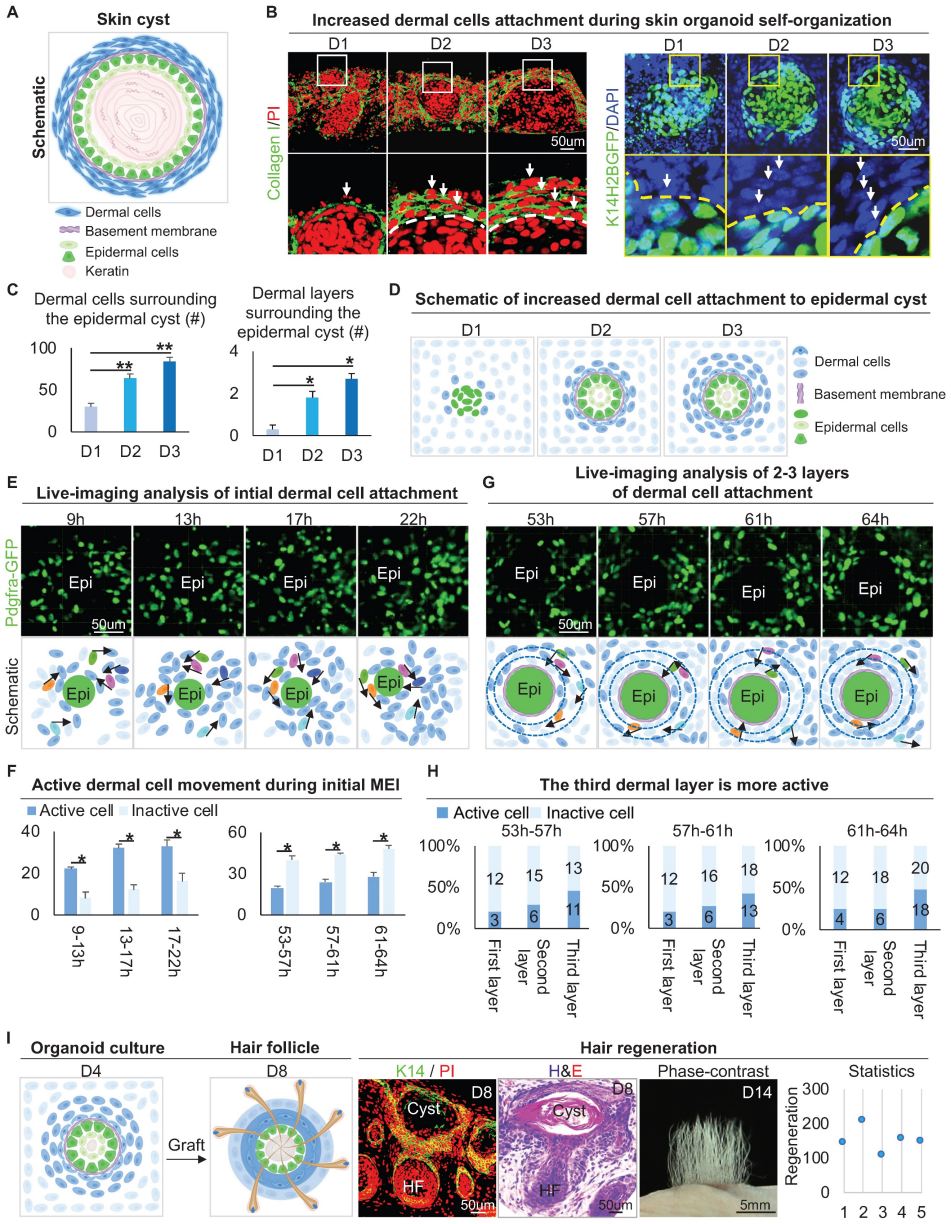
** Attachment of dermal cells to the epidermal cysts in skin organoid culture and hair regeneration upon transplantation.** A. Schematic of an epithelial cyst. B. Collagen I immunostaining and wholemount scanning of skin organoid cultures of K14H2BGFP transgenic mice cells show the layers of dermal cells surrounding the epidermal cyst from D1 to D3. Scale bars, 50 um. C. Statistical of the dermal cells (left) and dermal layers (right) surrounding the epidermal cyst. D. Schematic of increased dermal cell attachment in skin organoid from D1 to D3. E. Time-lapse live imaging of Pdgfra-EGFP mouse cells shows dermal cell initial attachment during 9-22 h. Schematic of dermal cell initial attachment and movement during 9-22 h. Dark and light blue represent active and inactive cells, respectively. The analysis was based on the movies published in our previous study 1. F. Statistical of the active or inactive number of dermal cells during 9-22 h and 53-64 h. G. Time-lapse live imaging of Pdgfra-EGFP mouse cells shows dermal cells move around the epidermal cyst during 53-64 h. Schematic of dermal cell movement during 53-64 h. The analysis was based on the movies published in our previous study 1. H. Statistical of the active or inactive number of dermal cells in the 1-3 layers of the dermal layers during 9-22 h and 53-64 h. N ≥ 3, **p < 0.01, *p < 0.05, and # no significant change. I. Schematic of hair regeneration from D4 skin organoid (left panels 1 and 2); K14 immunostaining and H&E staining show the cyst structure formation in the hair regeneration (mid-panels 1 and 2); hair regeneration from the skin cysts, and statistical of the number of regenerated hair follicles (right panels 1 and 2).

The epithelial cyst consists of basal cells, suprabasal cells, and keratin debris, and most of the epithelial cysts are surrounded by a basement membrane attached with layers of dermal cells under physiological or pathological conditions (Figure 1A). H&E staining and Vimentin immunostaining showed that epithelial cysts in mouse skin are surrounded by layers of dermal cells (Figure S1A). We also observed that the skin organoid formed cysts from dissociated cells to aggregate from D0 to D3 culture 1. Interestingly, Collagen I immunostaining and wholemount scanning of organoid cultures of K14H2BGFP transgenic mice skin cells showed that the layers of dermal cells surrounding the epidermal aggregate are significantly increased from D1 to D3 (Figure 1B-C), and this is verified by K14 immunostaining and H&E staining (Figure S1B-C). Further immunostaining showed that the dermal cells are attached to the basement membrane which is positive for Laminin and Collagen IV (Figure S1D). Consistent with our previous finding, we observed the epidermal aggregate polarizes from D1 to D3, which is characterized by P-cadherin immunostaining (Figure S1E). Meanwhile, Collagen VII immunostaining showed anchoring fibril between the epidermal and the dermal cells from D1 to D3. Thus, we characterized that the skin cyst mainly consists of epidermal aggregate, with dermal cells attachment and a basement membrane lying between (Figure 1D). These results suggest increased MEI from D1 to D3 during skin organoid culture.

To observe how dermal cells initiate MEI, we analyzed our 4D movies from live imaging of Pdgfra-GFP mouse cells during 9-22 h and 53-64 h in skin organoid cultures 1, which represent the periods of initial dermal cell attachment and 2-3 layers of dermal cell attachment, respectively. Most of the dermal cells move quickly during 9-22 h, only with 3-4 dermal cells initially attaching to the epidermal cyst (Figure 1E-F). When 2-3 layers of dermal cells surround the epidermal cells, we observed that dermal cells still move around the epidermal cyst, with the outermost dermal cells moving most actively during 53-64 h (Figure 1G-H). In addition, by analyzing the live imaging movies of FVB-GFP mouse cell behavior which shows the whole cell morphology, we observed the dermal cells not only circle the epidermal cyst but also stretch the epidermal cells during 52-57 h in skin organoid cultures 1 (Figure S1F). These results suggested that dermal cell movement triggers the initial MEI event, and may generate the stretch force to the epidermal cells.

It is known that MEI is required for the development of hair follicles during embryogenesis 37. Indeed, when grafting the D4 cultured skin organoids onto the back of nude mice, we observed that the hair follicles form from the skin cysts (Figure 1I). Interestingly, when adult mouse cells are used to do organoid cultured 1, dermal cells are not attached to the epidermal cyst, as demonstrated by immunostaining for Vimentin and K14 (Figure S1G). When grafting the D4 adult skin organoid cultures (no dermal cells attachment) onto the back of nude mice, we observed that the hair follicles did not form (Figure S1G). To further confirm that MEI is important for hair regeneration, we performed a recombination assay, in which newborn epidermal cells plus adult dermal cells (NE+AD) and newborn dermal cells plus adult epidermal cells (ND+AE) were mixed and cultured. Wholemount staining and transplantation assay showed that the ND+AE group undergoes the initial MEI event more similar to that of the newborn mouse cells, and leads to hair regeneration, but the NE+AD did not (Figure S1H). These results suggested that MEI in the skin cyst potentiates skin organoids to regenerate hair follicles upon transplantation.

### Native dermal cells-derived mechanical force regulates dermal cell attachment

**Figure 2:**
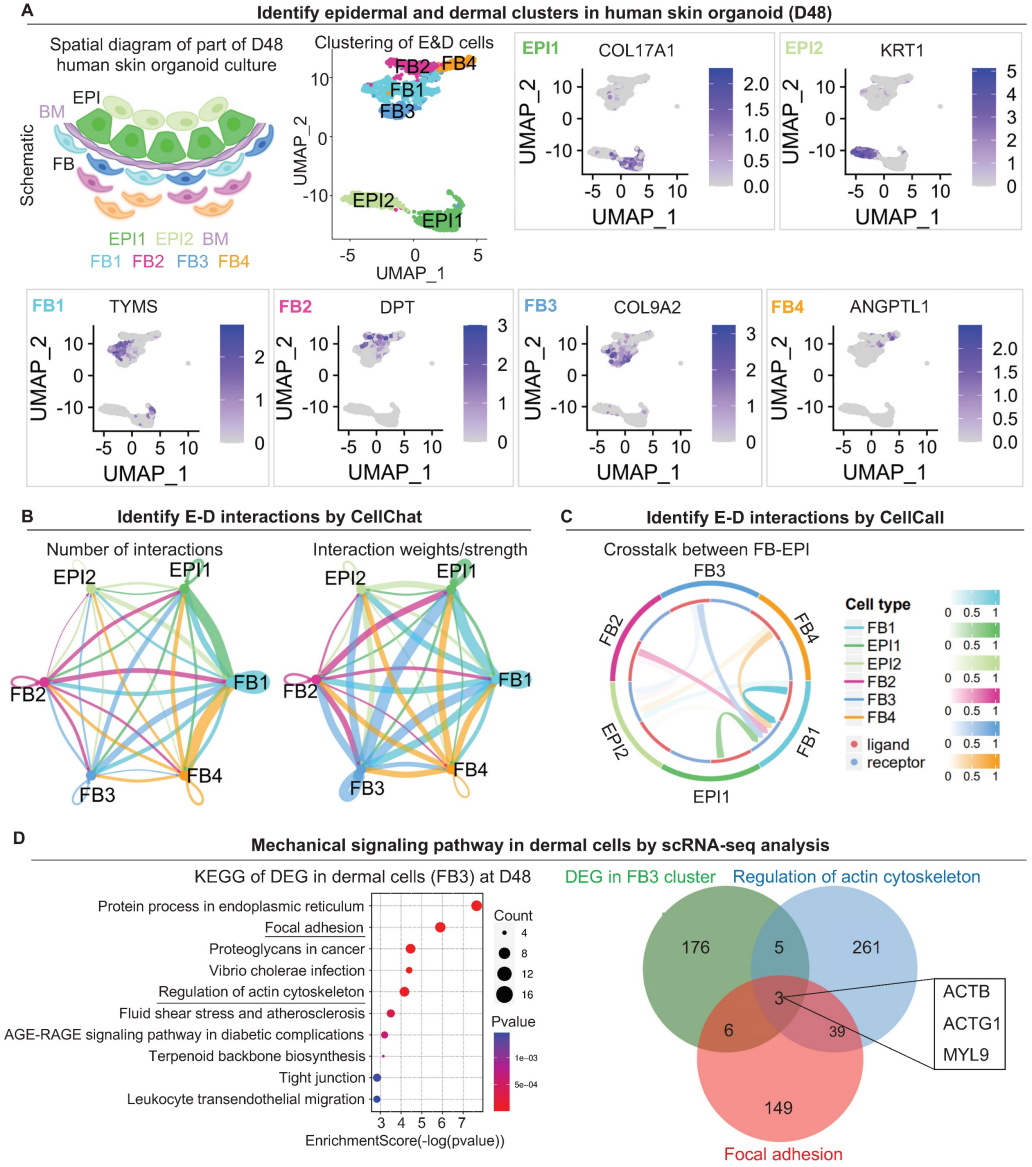
** scRNA-seq shows epidermal and dermal cell interactions and mechanical signaling pathways in dermal cells in human skin organoid culture.** A. Schematic of human skin organoid at D48. UMAP plots of epidermal and dermal clusters by unbiased clustering. B. CellChat analysis of epidermal and dermal cell interactions. C. CellCall analysis of epidermal and dermal cell interactions. D. KEGG analysis shows the regulation of the actin cytoskeleton in dermal cells (FB3) (left); Venn diagram shows overlapping genes between the Regulation of the actin cytoskeleton pathway, Focal adhesion pathway, and FB3 cluster.

Our previous study focused more on epidermal cell differentiation during the polarization stage 1. How dermal cells behave during this stage remains elusive. To explore the mechanisms by which the dermal cells attach to the epidermal cyst, we analyzed the single-cell RNA sequencing (scRNA-seq) data performed for the D48 human skin organoid with a skin cyst that has the epithelial and mesenchymal components 3 (Figure 2A). By isolating the epidermal and dermal cells, we identified 6 subtypes of cells through unbiased clustering. Uniform Manifold Approximation and Projection (UMAP) plots show two sub-clusters of epidermal cells (EPI1 and EPI2) and four types of dermal fibroblasts (FB1, FB2, FB3, and FB4), with each cell type having its own molecular identity (Figure 2A). CellChat 35 and CellCall 36 analysis revealed interactions between two sub-clusters of epidermal cells (EPI1 and EPI2) and four types of dermal fibroblasts (FB1, FB2, FB3, and FB4) (Figure 2B-C). The number of interactions and interaction weights/strength are strong between basal epidermal cells (EPI1) and FBs (1 & 3) (Figure 2B). Kyoto Encyclopedia of Genes and Genomes (KEGG) analysis reveals that the mechanical signaling pathways were highly enriched in FB1 and FB3, but less in other epidermal or dermal cell sub-clusters (Figure S2A). Particularly, KEGG analysis shows that both the Focal adhesion and Regulation of the actin cytoskeleton are significantly enriched in FB3. Venn diagram shows that genes related to the regulation of actin cytoskeleton are also enriched in Focal adhesion (ACTB, ACTG1, MYL9) (Figure 2D). It is well known that MYL9 (myosin regulatory light polypeptide 9) and MYH9 (myosin heavy chain 9) interacts with actins in the cytoplasm to take part in different cellular processes such as cell motility, cell migration and cell adhesion 38. UMAP plot shows MYH9 is expressed in epidermal and dermal clusters, and MYL9 is expressed in dermal cell (Figure S2B). Immunostaining showed that MYH9 is highly expressed in the junction of dermal and epidermal cells in the organoids (Figure S2C). Immunostaining showed that MYL9 is distributed in almost all cells, and phosphorylated MYL9 (p-MYL9, Thr19/Ser20) is highly expressed at the junction of dermal-epidermal cells and peripheral dermal cells in the organoids (Figure S2D).

**Figure 3:**
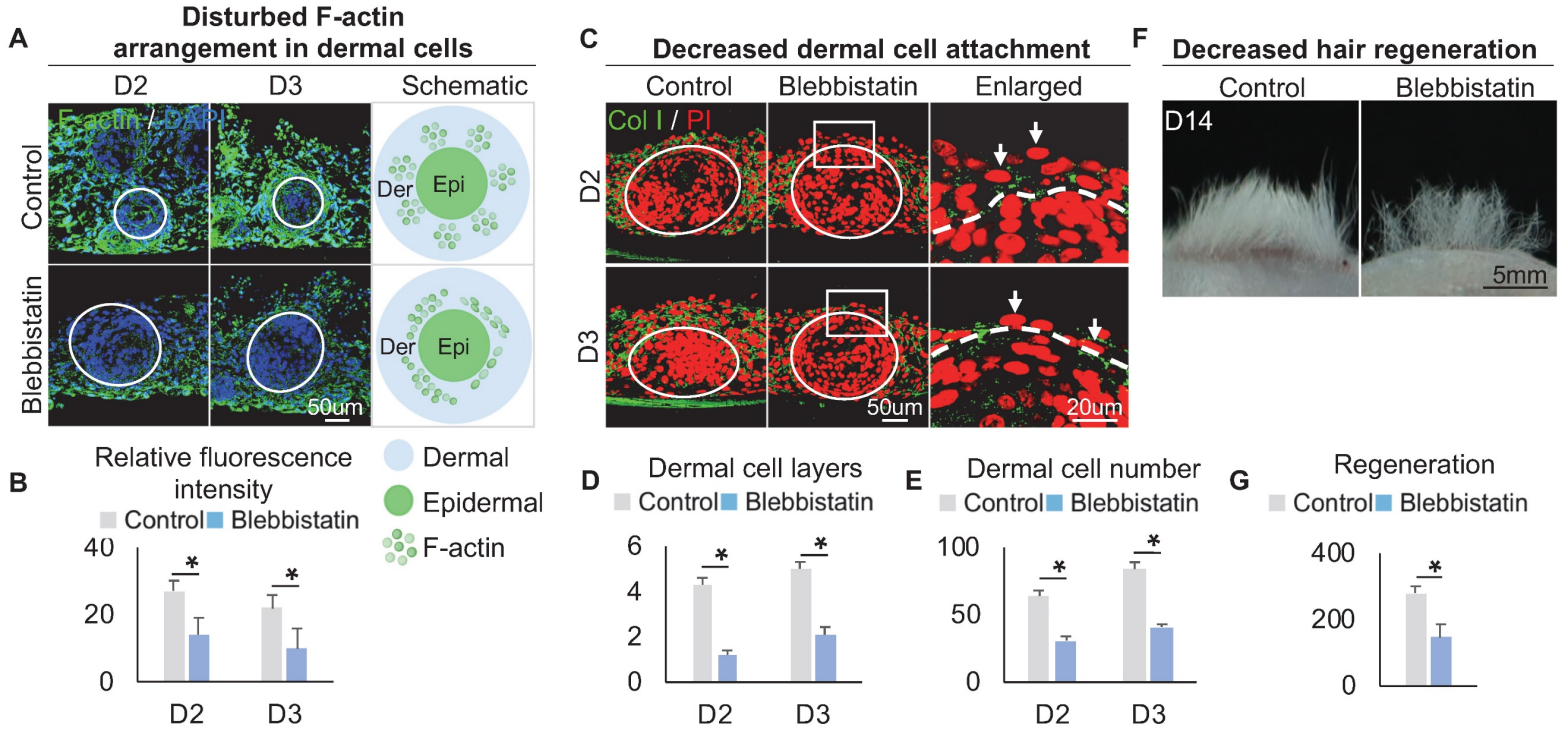
** The decreased dermal cell attachment in skin organoid culture after treatment of Blebbistatin.** A. F-actin immunostaining and schematics show F-actin arrangement in dermal cells after treatment of Blebbistatin. B. Statistics of the relative fluorescence intensity between the control group and the Blebbistatin group. C. Collagen I immunostaining shows dermal cell attachment to the epidermal cyst after treatment of Blebbistatin. D. Statistics of the dermal cell layers surrounding the epidermal cyst after treatment of Blebbistatin. E. Statistics of the dermal cell numbers surrounding the epidermal cyst after treatment of Blebbistatin. F. Hair regeneration after grafting skin organoid cultures treated with Blebbistatin onto the back of the nude mice. G. Statistics of hair regeneration. P < 0.05, n = 3. N ≥ 3, *p < 0.05, and # no significant change.

Within these two KEGG terms, F-actin, which provides mechanical force for cell movement, is also highly expressed in FB3 which is verified by immunostaining (Figure 3A), indicating that FB3 is the main dermal cluster surrounding the epidermal aggregate. When applied Blebbistatin, a myosin II inhibitor 39, 40 that ablates mechanical forces, the expression of the cytoskeleton was significantly decreased in the dermal cells surrounding the epidermal aggregate, compared to the control group which shows more spread and extended cytoskeleton (Figure 3A-B). This indicates that dermal cells can generate native mechanical forces during organoid culture. To further confirm that mechanical force is generated in dermal cells, immunostaining showed that the addition of Blebbistatin resulted in significantly decreased MYH9, MYL9 and p-MYL9 expression in the junction of dermal and epidermal cells (Figure S3A-B). Immunostaining for Collagen I and K14 showed that the addition of Blebbistatin resulted in significantly decreased attachment of dermal cells surrounding the epidermal aggregate compared to the control (Figure 3C-E, Figure S3C), with disrupted basal layer formation as indicated by Collagen XVII immunofluorescence staining (Figure S3D). Meanwhile, Live-Dead staining showed that Blebbistatin did not affect survival in the organoids (Figure S3E). Transplantation of Blebbistatin-treated culture into the nude mice resulted in dramatically decreased hair regeneration (Figure 3F-G). Interestingly, H&E staining shows the hair cycle in the skin regenerated by the transplantation of Blebbistatin-treated cultures (Figure S3F). These results imply that native mechanical force derived from dermal cells is necessary for dermal cell attachment and influences prospective hair regeneration.

### Calcium regulates the arrangement of the dermal cytoskeleton to influence dermal cell attachment

**Figure 4:**
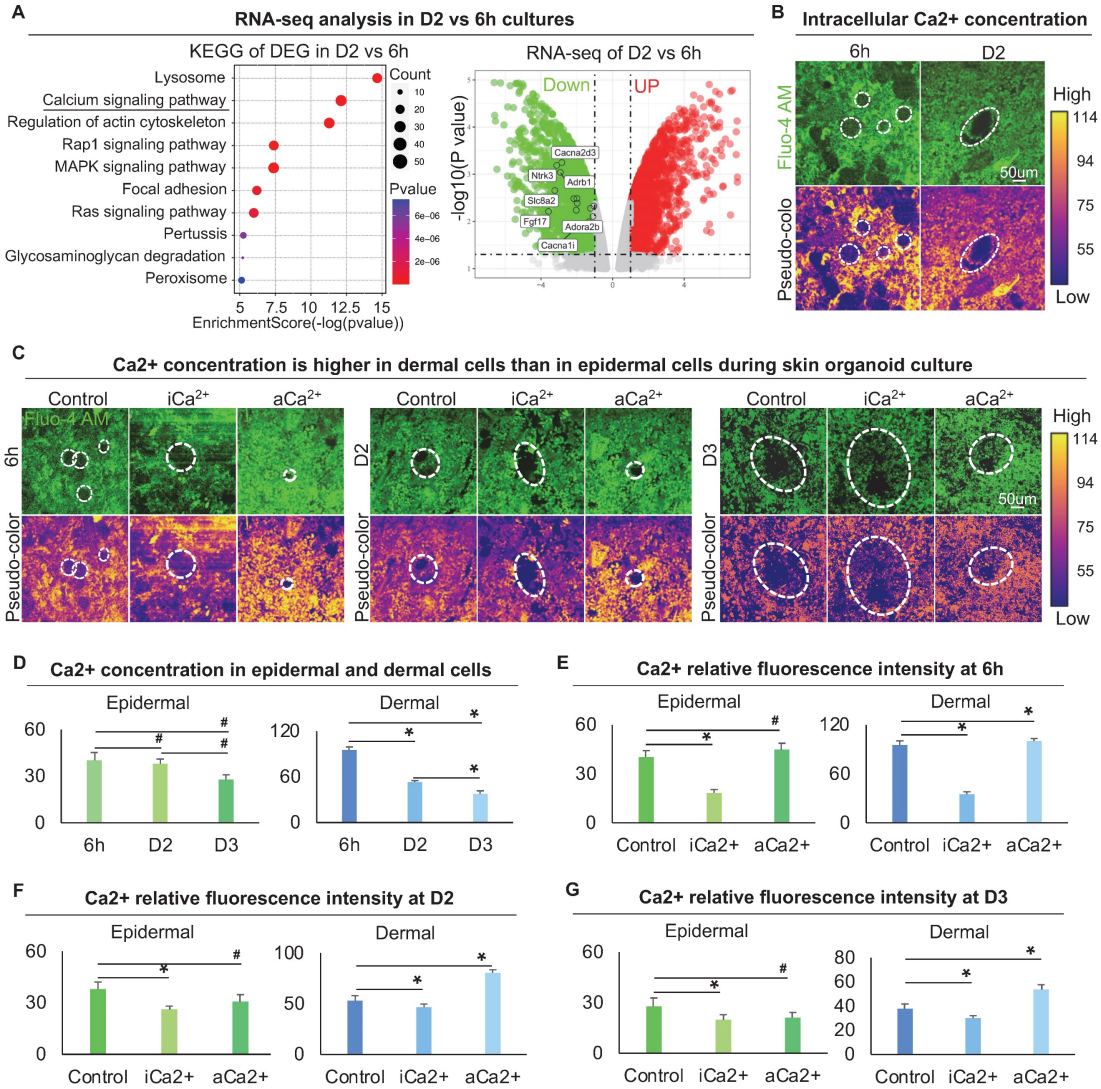
** Characterization of the intracellular calcium concentration between epidermal and dermal cells.** A. RNA-seq compares gene expression between 6 h and D2 skin organoid culture. KEGG analysis shows the Ca2+ signaling pathway enriched in differentially expressed genes (DEGs) of D2 vs 6 h cultures (left); Volcano plot shows Ca2+ signaling pathway genes expression in D2 vs 6 h skin organoid cultures (right). B. Fluo-4 AM fluorescent probe detected Ca2+ concentration at 6 h and D2. C. Fluo-4 AM fluorescent probe detected Ca2+ concentration within Ca2+ inhibitor (ruthenium red, iCa2+) or Ca2+ activator (thapsigargin, aCa2+) cultures at 6 h, D2 and D3. D. Statistics of intracellular calcium concentration in epidermal and dermal cells at 6h, D2 and D3. E. Statistics of intracellular calcium concentration in epidermal and dermal cells in iCa2+ and aCa2+ groups at 6 h. F. Statistics of intracellular calcium concentration in epidermal and dermal cells in iCa2+ and aCa2+ groups at D2. G. Statistics of intracellular calcium concentration in epidermal and dermal cells in iCa2+ and aCa2+ groups at D3. N ≥ 3, *p < 0.05, and # no significant change.

To explore the regulator of the cytoskeleton that controls dermal cell attachment to the epidermal cyst, we analyzed our bulk RNA-sequencing (RNA-seq) data (GSE86955) for 6 h and D2 cultures, which represent the time points with or without dermal cell attachment, respectively. KEGG signaling pathway and Volcano plot analysis showed that the differentially expressed genes (DEGs) were highly enriched in Lysosome, Calcium signaling pathway, and Regulation of actin cytoskeleton, etc. (Figure 4A). Calcium signaling is ubiquitous in eukaryotic cells and modulates many cellular events, including regulation of cell retraction due to cytoskeletal rearrangement and cell migration due to adherens junction disassembly 41, 42. Therefore, we speculated that the differentially expressed calcium signaling between D2 and 6 h cultures may affect cytoskeletal rearrangement which further influences dermal cell attachment and MEI. Volcano plot and hierarchical clustering showed that genes related to the calcium signaling pathway were significantly down-regulated at D2 vs 6 h (Figure 4A, Figure S4A). Using a Fluo-4 AM fluorescent probe, we detected that the intracellular calcium concentration is decreased from 6 h to D2 culture (Figure 4B). The intracellular calcium concentration is higher in dermal cells than in epidermal cells at 6 h and D2, indicating that intracellular calcium may affect the behavior of dermal cells more than epidermal cells (Figure 4B). To confirm that whether mechanical force regulates the calcium concentration, Fluo-4 AM fluorescent probe detection assay showed that the addition of Blebbistatin did not affect calcium concentration in both epidermal and dermal cells during skin organoid culture (Figure S4B).

To explore the effect of calcium on dermal cell behavior, we treated skin organoid cultures with ruthenium red, a calcium inhibitor 43, 44, or with thapsigargin, a calcium activator 45. Using the Fluo-4 AM fluorescent probe, we further observed that dermal cells are more responsive to intracellular calcium (Figure 4C-G). Nevertheless, activating calcium eventually resulted in a smaller epidermal aggregate in size at 6 h, D2, and D3 (Figure 4C-G), indicating that change in dermal cells influences epidermal cell behavior.

**Figure 5:**
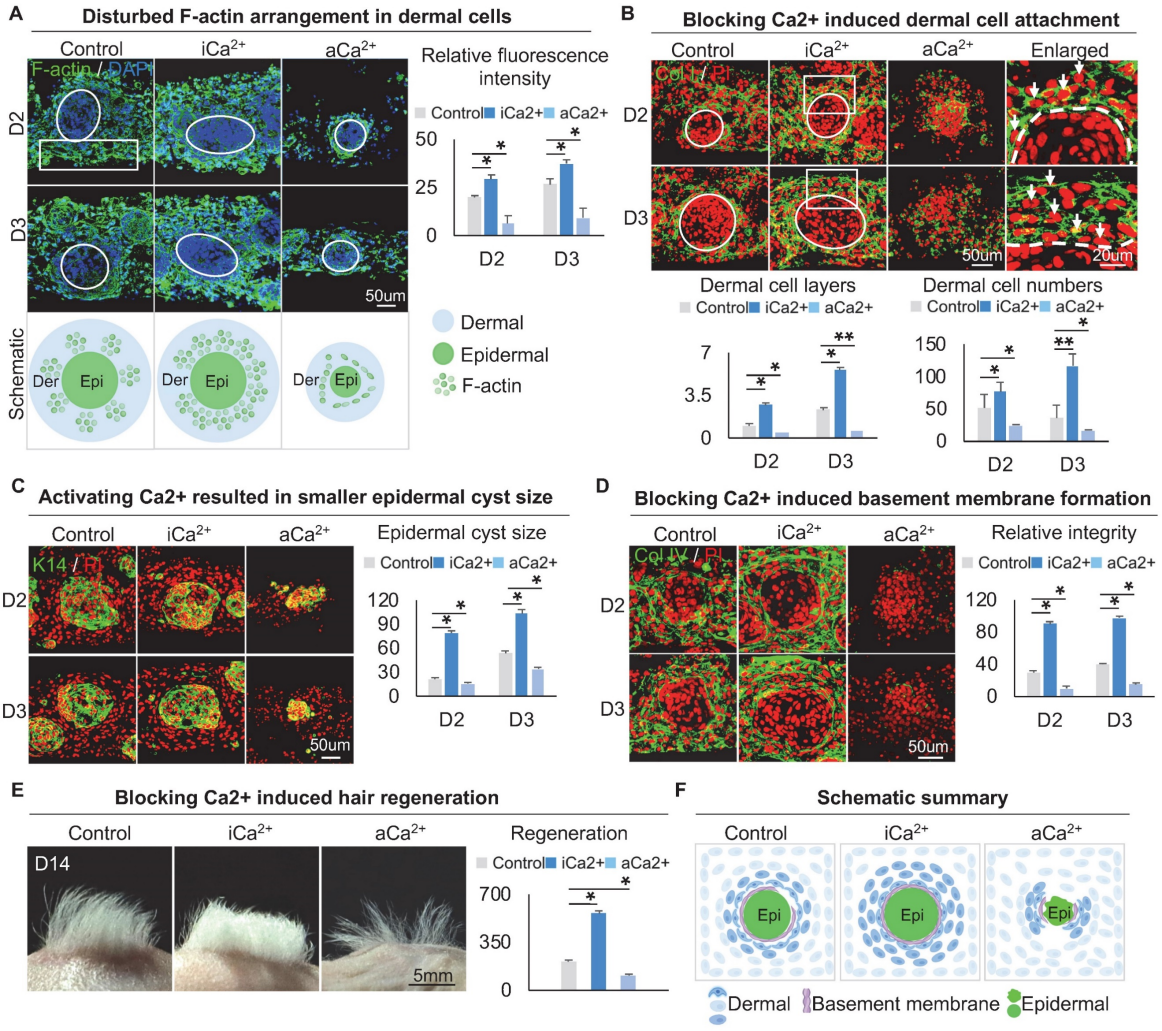
** Calcium regulates the arrangement of the dermal cytoskeleton to influence dermal cell attachment.** A. F-actin immunostaining, statistical analysis, and schematic show F-actin arrangement after treatment with thapsigargin or ruthenium red. B. Collagen I immunostaining and statistical analysis show more dermal cell attachment after blocking calcium signaling in D2 and D3 cultures. C. K14 immunostaining and statistical analysis show a larger epidermal cyst in size after blocking or activating the calcium signaling. D. Collagen IV immunostaining and statistical analysis show that blocking the calcium signaling promotes basement membrane formation. E. Phase-contrast microscope and statistical analysis show hair regeneration after grafting skin organoid cultures treated with thapsigargin or ruthenium red. N ≥ 3, **p < 0.01, *p < 0.05, and # no significant change. F. Schematic of skin organoid culture at D3 after treatment with thapsigargin or ruthenium red.

F-actin immunostaining showed that, compared with the dermal cells which exhibited a highly-branched shape and long filopodia in the control group, the dermal cells in the calcium signaling-inhibited group displayed a polygonal morphology with fewer branch points and a thick actin fiber formation whereas the dermal cells in the calcium signaling-overactivated group displayed a thin actin fiber formation (Figure 5A, S5A). Meanwhile, immunostaining showed that blocking calcium did not affect MYH9 expression, but promoted p-MYL9 expression in dermal cells (Figure S5B-C). These results suggested that lower calcium concentration modulated dermal cells to exhibit thicker actin fibril formation, thereby enhancing interactions between dermal cells surrounding the epidermal aggregate.

We then explored if calcium signaling influences dermal cell attachment to the epidermal aggregate. Immunostaining for Collagen I, K14, and E-cadherin showed that blocking calcium signaling induces more dermal cell attachment whereas activating calcium signaling blocks dermal attachment (Figure 5B-C, Figure S5D). In addition, blocking calcium signaling resulted in a larger epidermal cyst in size whereas activating calcium signaling resulted in a smaller epidermal cyst in size (Figure 5C, Figure S5E), with more expression of P-cadherin which represents a higher multipotency (Figure S5F). Interestingly, immunostaining for Collagen IV and Laminin showed that blocking the calcium signaling promotes basement membrane formation whereas activating calcium signaling blocks basement membrane formation (Figure 5D, Figure S5G), suggesting that blocking the calcium signaling enhances MEI. Transplantation of calcium signaling-blocked organoids onto the back of nude mice resulted in significantly more hairs regeneration (Figure 5E). H&E staining shows the hair cycle in the skin regenerated by the transplantation of the calcium signaling-blocked or activated cultures (Figure S5H). These results suggest that intracellular calcium affects dermal cell attachment at the periphery of skin cyst by regulating the arrangement of the dermal cytoskeleton, thereby affecting the MEI (Figure 5F).

### Mechanical stretching inhibits dermal cell attachment but induces Piezo1 in the epidermal cyst

**Figure 6:**
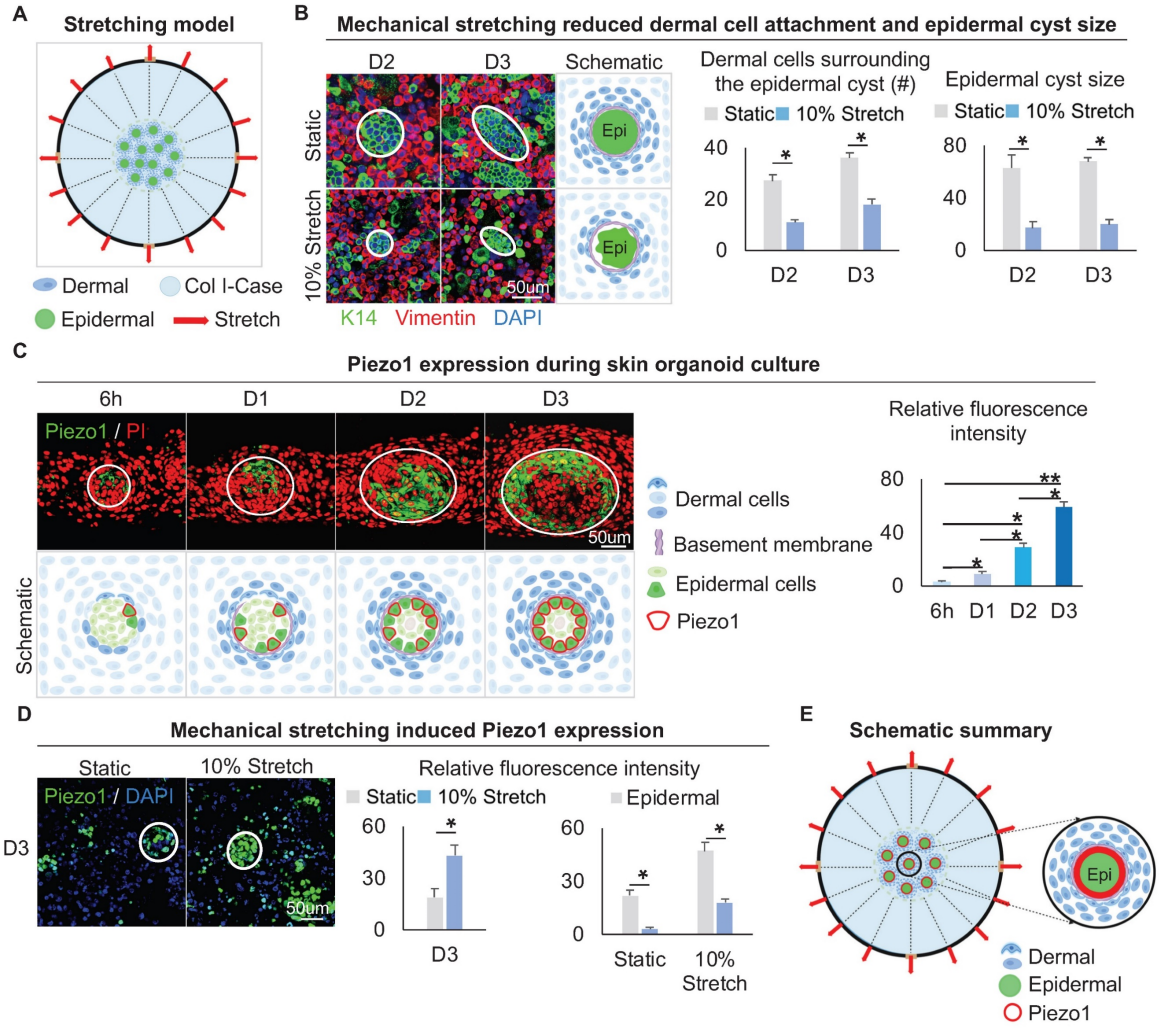
** Mechanical stretching inhibits dermal cell attachment but induces Piezo1 expression in the epidermal cyst.** A. Schematic of the flexcell mechanical stretching model. B. Wholemount immunofluorescence staining of Vimentin and K14 shows dermal cell attachment and epidermal cyst size after mechanical stretching (left). Statistics of the dermal cells surrounding the epidermal cyst (middle) and epidermal cyst size (right) after flexcell mechanical stretching. C. Piezo1 immunostaining and quantification show Piezo1 expression during skin organoid culture. D. Piezo1 immunostaining and quantification show that mechanical stretching induced Piezo1 expression. N ≥ 3, *p < 0.05, and # no significant change. E. Schematic of the Piezo1 expression after mechanical stretching with flexcell.

The above results led us to speculate that the native mechanical force generated from the dermal cell movement may exert a stretching force on the adjacent epidermal cells. Therefore, we explored how the stretching force influences the MEI during skin cyst formation. Using the flexcell mechanical stretching model 46, we applied stretching force to the skin organoid culture for 12 h. (Figure 6A). Double immunofluorescence staining of Vimentin and K14 showed that the dermal cells surrounding the epidermal cyst are looser upon mechanical stretching compared to the static control, and the size of the epidermal cysts is reduced (Figure 6B), implying the efficacy of application of stretching force in skin organoid culture. Similarly, immunostaining for Collagens type I and IV showed that stretching force reduces Collagens expression compared to the static control (Figure S6A).

We then examined the expression of Piezo1, which is a piezoelectric force-sensitive ion channel factor that can determine cell fate by sensing mechanical stretching force 47. UMAP plot shows Piezo1 is expressed in epidermal and dermal clusters (Figure S6B). Immunofluorescence staining showed that Piezo1 was preferentially expressed in the basal layer of the epidermal aggregate at D3, and its expression is increased from 6 h to D3 during self-organization of skin organoid (Figure 6C). This suggests that the basal layer may be subjected to the surrounding dermal cells-derived stretching force, which triggers the activation of Piezo1 in the basal cells. We performed an experiment by applying 5%, 10%, and 15% mechanical stretch to organoid cultures, and observed that the stretch-induced Piezo1 activation was positively correlated with the strength. However, the application of high-strength (15%) stretch significantly affected the dermal cell attachment to the epidermal aggregate. This may be due to an external mechanical factor (high mechanical stretch) but not stretch-induced Piezo1 activation. So, 10% mechanical stretch can induce Piezo1 activation without affecting the attachment of dermal cells to the epidermal aggregate (Figure S6C). Immunofluorescence staining and quantitative RT-PCR showed that the application of 10% mechanical stretching enhanced the expression of Piezo1 in both epidermal and dermal cells (Figure 6D, Figure S6D). We further explored whether mechanical force affects Piezo1 expression or intracellular calcium concentration affects Piezo1 expression, and immunofluorescence staining showed that calcium concentration did not affect the expression of piezo1 (Figure S6E). Together, these results suggested that mechanical stretching force generated by the dermal cells can activate the mechanosensitive channel factor Piezo1 in the epidermal basal cells during organoid culture (Figure 6E).

### Epidermal Piezo1 in turn drives MEI to regulate dermal cell attachment

**Figure 7:**
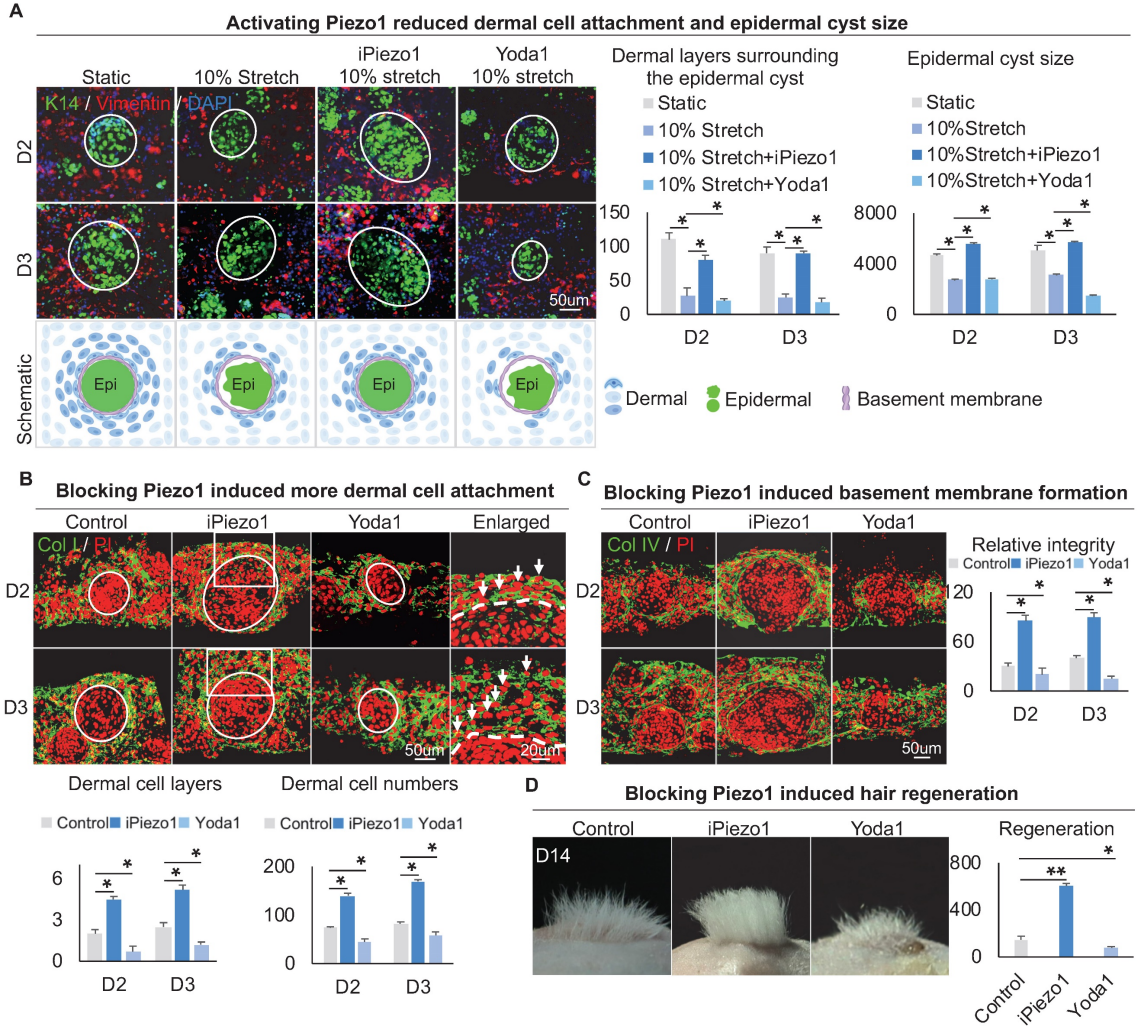
** Epidermal Piezo1 drives MEI to regulate dermal cell attachment.** A. Wholemount immunofluorescence staining of Vimentin and K14 (left) shows dermal cell attachment and epidermal cyst size after mechanical stretching or treatment with Piezo1 inhibitor (GsMTx4 TFA) or Piezo1 activator (Yoda1). Statistics of the dermal cells surrounding the epidermal cyst (middle) and epidermal cyst size (right). B. Collagen I immunostaining and statistics show more dermal cell attachment surrounding the epidermal cyst after blocking Piezo1. C. Collagen IV immunostaining and statistics show that blocking Piezo1 promotes basement membrane formation. Hair regeneration after grafting skin organoid cultures treated with GsMTx4 TFA or Yoda1 onto the back of the nude mice. Phase-contrast microscope and statistical analysis show hair regeneration after grafting skin organoid cultures treated with GsMTx4 TFA or Yoda1. N ≥ 3, **p < 0.01, *p < 0.05, and # no significant change.

To explore the effect of Piezo1 on MEIs during skin organoid self-organization, we treated skin organoid cultures with GsMTx4, a Piezo1 inhibitor 43, 44, or with Yoda1, a Piezo1 activator 45. Compared to the static group and mechanical stretching-treated group, wholemount staining showed that the Yoda1 addition and mechanical stretch resulted in less dermal cell attachment (Figure 7A). However, with the addition of GsMTx4 and mechanical stretch, dermal cell attachment was restored (Figure 7A). Interestingly, activation of Piezo1 also resulted in smaller-sized epidermal cyst formation (Figure 7A, Figure S7A), with disrupted basal layer formation as indicated by Collagen XVII and P-cadherin immunofluorescence staining (Figure S7C-D). Immunofluorescence staining of Collagen I and K14 showed that inhibition of Piezo1 resulted in more dermal cell attachment (Figure 7B, Figure S7A). Immunostaining for Collagen IV and Laminin showed that activating Piezo1 inhibited basement membrane formation (Figure 7C, Figure S7B). Inhibition of Piezo1 resulted in more hair regeneration upon transplantation to the back of nude mice (Figure 7D). H&E staining shows the hair cycle in the skin regenerated by the transplantation of the Piezo1-blocked or activated cultures (Figure S7E). Last, we also detected the effect of Piezo1 on calcium concentration, and observed that the calcium concentration is significantly decreased in the iPiezo1 group compared to the control (Figure S7F). These results suggest that the mechanical stretching force-induced epidermal Piezo1 negatively regulates dermal cell attachment.

## Discussion

Organoids are 3D-structured cultures that can form mini-organs with similar architecture to those of the physiologically-developed ones. A common question in the organoid field is how the stem or progenitor cells self-organize to form a cyst structure that is primed to generate specific mini-organs. During skin organoid morphogenesis 1, we also observed the formation of skin cysts which include epidermal cells, dermal cells, and a basement membrane lying between, and they produce hair follicles upon transplantation (Figure 1E). In this study, we used our established skin organoid model to address the above question. We showed that dermal cells generate initial mechanical force that triggers initial MEI during skin organoid development. This potentiates skin organoids to form a skin cyst which can regenerate hair follicles upon transplantation. Particularly, we focused on the dermal cell behavior during this process.

Mechanical force is largely involved in regulating cellular behaviors in living life 16, 48. For example, the contraction of dermal progenitor cells triggers the mechanosensitive activation of b-catenin in adjacent epidermal cells, initiating the feather follicle morphogenesis program in embryonic chicken skin 49. Our findings show that the dermal cells-derived mechanical force is critical for dermal cells to attach to the epidermal aggregate and drives initial MEI during skin organoid development. This process involves stepwise and cascaded mechano-chemical cross-talks.

First, generation of the initial mechanical force. We observed that the dermal cells circle the adjacent epidermal aggregate by live imaging and wholemount staining. Why do dermal cells circle the epidermal aggregate? It is reported that the dermal cells can move in a platform with Laminin expression 50. We indeed observed that the epidermal aggregate expresses Laminin during the development of skin organoid formation 1. This may lead to dermal cell movement which generates a contraction force to themselves (as indicated by the F-actin staining) and a stretching force to the adjacent epidermal cells (as indicated by the live imaging analysis).

Second, dermal cell movement-generated force triggers differential distribution of intracellular calcium signaling in dermal cells, with a higher intracellular calcium concentration in dermal cells surrounding epidermal aggregate and lower in other dermal cells. This may lead to the differential cellular behaviors between the major two dermal fibroblast groups. We focused on studying the dermal fibroblasts surrounding the epidermal aggregate and found that the differential distribution of intracellular calcium signaling influences the arrangement of the cytoskeleton in those dermal cells. How does intracellular calcium signal influence dermal cell attachment? A previous study showed that a lower calcium concentration (0.5 mM) regulates the adhesion of osteoblasts through the integrin α5β3 / Rho A / Cytoskeleton signaling pathway, while a higher calcium concentration (1.2 mM) activates YAP/TAZ to inhibit the adhesion of osteoblasts 51. Thus we can speculate that a high calcium concentration in the dermal cells surrounding the epidermal aggregate weakens the interaction between α-actinin and actin filaments that results in short-term depolymerization of the cytoskeleton that inhibits dermal cell attachment to the epidermal aggregate.

Third, higher dermal calcium concentration triggers Piezo1 expression in adjacent epidermal cells. Why do the epidermal cells but not the dermal cells themselves sense the mechanical force-induced high calcium concentration? It has been established that Piezo1 serves as a sensor and transducer of mechanical stimulus in invertebrate embryos 29, 52. In the vast majority of systems, Piezo1 is activated by cell stretching force but not contraction force 32, 47, 53. The derma cells generate contraction force for themselves but give stretching force to the adjacent epidermal cells in organoid culture. Our Flexcell-stretching experiment also verified that the additional cell stretching can enhance the activation of Piezo1 in epidermal cells. These suggest that the differential mechanical forces impact different cellular and molecular behaviors in organoid culture. Nevertheless, we cannot ignore the force that may be generated by epidermal cells which induce Piezo1 activation. In skin organoid culture, epidermal aggregates continue to grow before reaching a maximal size (~350 cells on average). When the cell density is large, random small-scale fluctuations between individual epidermal cells will be amplified to form a large mechanical stretching force 49, which may also trigger Piezo1 expression. This will be studied in the future.

During development and regeneration, MEI enables the formation of skin appendages, such as follicles (hair, feather, and spine) 6, 37 and glands (salivary, mammary, and sweat). Mesenchymal cells can alter the properties and cell behavior of epithelial cells whereas epithelial cells also alter the properties and cell behavior of mesenchymal cells. For example, mesenchymal cells specify epithelial cell fate by inducing them to form placodes which conversely promote dermal condensate formation during the morphogenesis of hair or feather follicles 54.

Skin organoids are mainly composed of epidermal and dermal cells. We showed that proper MEI during skin organoid morphogenesis *in vivo* is required for hair regeneration upon transplantation 1. It is intuitive to consider that this requires dermal-epidermal coupling during skin organoid development. Indeed, transplanting skin organoids with a larger number of dermal cells attached to the epidermal aggregate into the back of nude mice resulted in a greater amount of hair regeneration, confirming that MEI determines the outcome of organogenesis. Thus, our system also provides a platform to modulate MEI to induce hair regeneration using readily-obtained cells which are refractory to form hair follicles.

In summary, using interdisciplinary approaches, our study revealed that dermal cells-derived mechanical force drives initial MEI during skin organoid self-organization, which facilitates hair follicle regeneration upon transplantation. Such interactions between epithelial and mesenchymal are also observed in the development and regeneration of other organs. Thus, our skin organoid model can be used to investigate tissue mechanics, epithelial-mesenchymal interaction, stem cell biology, organogenesis, and drug screening. This work also paves a new way for studying the combination of mechanical-chemical coupling that drives tissue self-organization, not only in the skin but also in all other types of organoids.

## Supplementary Material

Supplementary figures and tables.

## Abbreviations

MEI: mesenchymal-epithelial interaction
3D: 3-dimensional
iPSC: induced pluripotent stem cells
MEIs: mesenchymal-epithelial interactions
Ca2+: calcium
ECM: extracellular matrix
D1: day 1
PFA: paraformaldehyde
PI: propidium iodide
H&E: hematoxylin and eosin
NE+AD: newborn epidermal cells plus adult dermal cells
ND+AE: newborn dermal cells plus adult epidermal cells
scRNA-seq: single-cell RNA sequencing
UMAP: uniform manifold approximation and projection
EPI: basal epidermal cells
FB: fibroblasts
KEGG: kyoto encyclopedia of genes and genomes
MYL9: myosin regulatory light polypeptide 9
MYH9: myosin heavy chain 9
p-MYL9: phosphorylated MYL9 (Thr19/Ser20)
RNA-seq: bulk RNA-sequencing
DEGs: differentially expressed genes
