# Supplementary material for: Mechanical force drives the initial mesenchymal-epithelial interaction during skin organoid development
Source: Theranostics. 2023 May 11;13(9):2930–45. doi: 10.7150/thno.83217 (PMC10240816; doi:10.7150/thno.83217)
Supplement: Supplementary file 1 — Supplementary figures and tables. [file thnov13p2930s1.pdf]

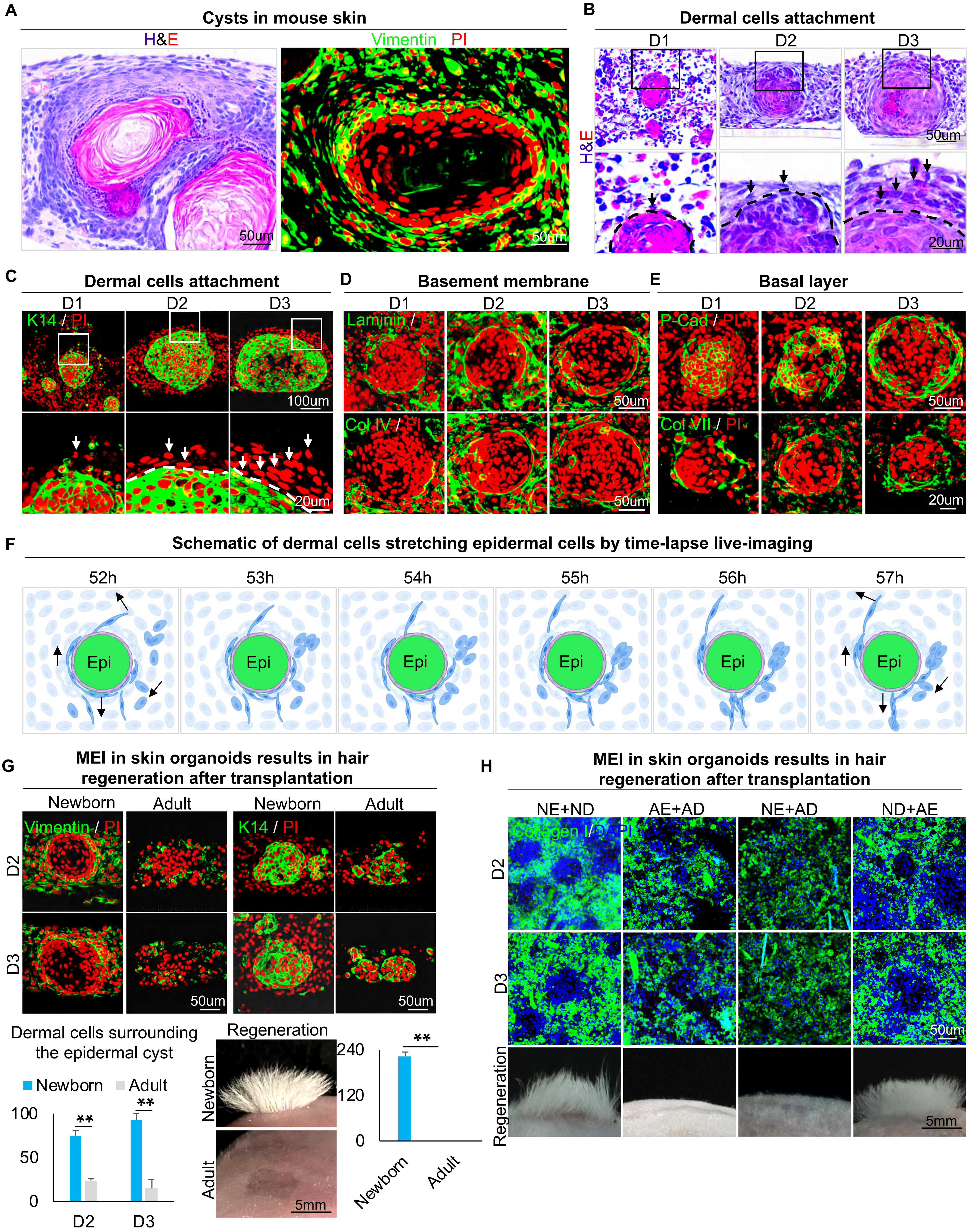

A
Cysts in mouse skin
H&E
Vimentin PI
50um
50um
B
Dermal cells attachment
D1
D2
D3
H&E
50um
20um
C
Dermal cells attachment
D1
D2
D3
K14 / PI
100um
20um
D
Basement membrane
D1
D2
D3
Laminin / PI
Col IV / PI
50um
50um
E
Basal layer
D1
D2
D3
P-Cad / PI
Col VII / PI
50um
20um
F
Schematic of dermal cells stretching epidermal cells by time-lapse live-imaging
52h
53h
54h
55h
56h
57h
Epi
G
MEI in skin organoids results in hair regeneration after transplantation
Newborn
Adult
Newborn
Adult
Vimentin / PI
K14 / PI
D2
D3
50um
50um
Dermal cells surrounding the epidermal cyst
Newborn
Adult
100
50
0
**
**
D2
D3
Regeneration
Newborn
Adult
5mm
240
120
0
**
Newborn
Adult
H
MEI in skin organoids results in hair regeneration after transplantation
NE+ND
AE+AD
NE+AD
ND+AE
D2
D3
Regeneration
50um
5mm

Figure S1. Skin cyst structure.

A. H&E staining and Vimentin immunostaining show that epithelial cysts in mouse skin are surrounded by layers of dermal cells.
B. H&E staining shows that the layers of dermal cells surrounding the epidermal aggregate are significantly increased from D1 to D3.
C. K14 immunostaining shows that the layers of dermal cells surrounding the epidermal aggregate are significantly increased from D1 to D3.
D. Laminin and Col IV immunostaining shows the dermal cells are attached to the basement membrane.
E. P-cadherin and Col VII immunostaining shows the epidermal aggregate polarizes from D1 to D3.
F. Schematic of dermal cell stretching epidermal cell by observing live-imaging of FVB-GFP mouse cells. The analysis was based on the movies published in our previous study (1).
G. Vimentin and K14 immunostaining shows that dermal cells attach to the epidermal cyst in newborn and adult mouse skin organoids, and hair regeneration from the newborn and adult organoid cultures. N = 3, **$p < 0.01$.
H. Wholemount immunostaining and transplantation assay show recombination of newborn and adult cells in organoid cultures results in differential hair regeneration. N, newborn cells. A, adult cells. Epi, E, epidermal cells. D, dermal cells.

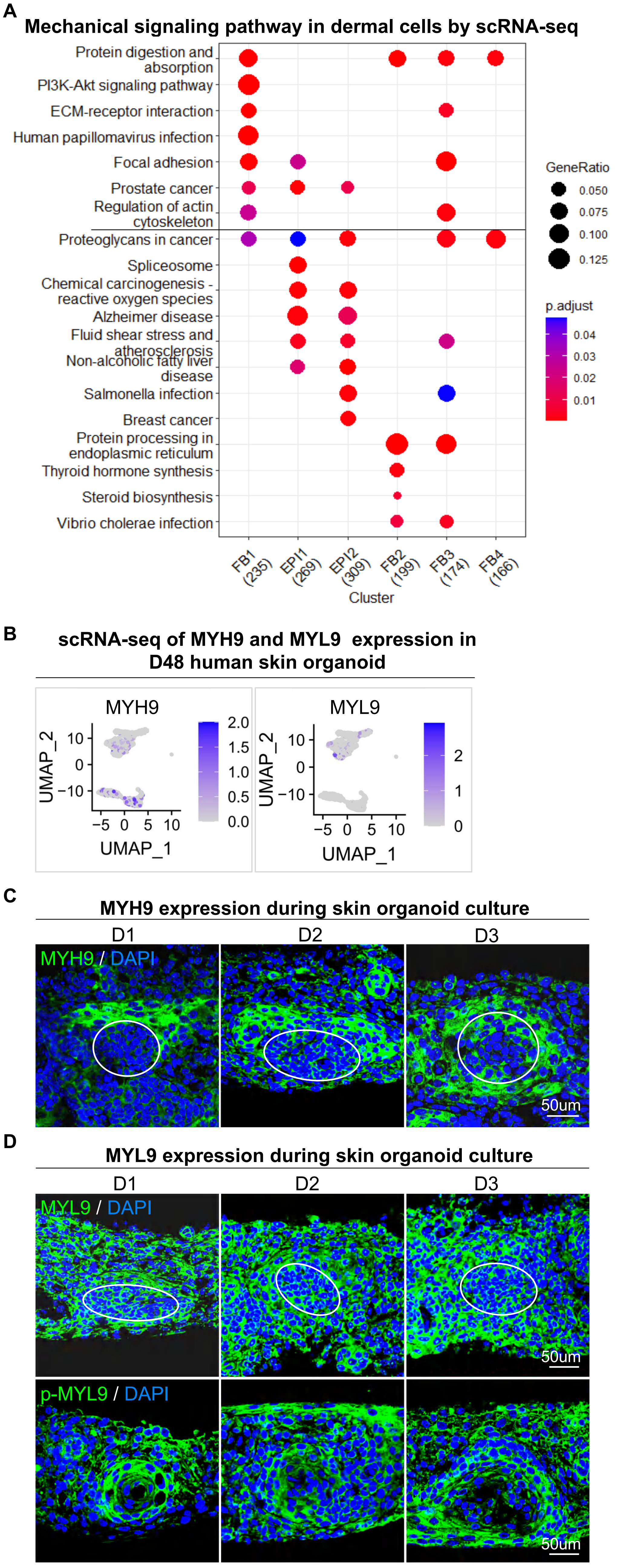
A
Mechanical signaling pathway in dermal cells by scRNA-seq
Protein digestion and absorption
PI3K-Akt signaling pathway
ECM-receptor interaction
Human papillomavirus infection
Focal adhesion
Prostate cancer
Regulation of actin cytoskeleton
Proteoglycans in cancer
Spliceosome
Chemical carcinogenesis - reactive oxygen species
Alzheimer disease
Fluid shear stress and atherosclerosis
Non-alcoholic fatty liver disease
Salmonella infection
Breast cancer
Protein processing in endoplasmic reticulum
Thyroid hormone synthesis
Steroid biosynthesis
Vibrio cholerae infection
FB1 (235)
EPI1 (269)
EPI2 (309)
FB2 (199)
FB3 (174)
FB4 (166)
Cluster
GeneRatio
0.050
0.075
0.100
0.125
p.adjust
0.04
0.03
0.02
0.01
B
scRNA-seq of MYH9 and MYL9 expression in D48 human skin organoid
MYH9
MYL9
UMAP_2
UMAP_1
10
0
−10
−5 0 5 10
2.0
1.5
1.0
0.5
0.0
2
1
0
C
MYH9 expression during skin organoid culture
D1
D2
D3
MYH9 / DAPI
50um
D
MYL9 expression during skin organoid culture
D1
D2
D3
MYL9 / DAPI
50um
p-MYL9 / DAPI
50um

Figure S2. scRNA-seq shows mechanical signaling pathway in dermal cells in human skin organoid culture.

A. KEGG analysis shows Regulation of the actin cytoskeleton in dermal cells (FB1 and FB3).

B. UMAP plots of MYH9 and MYL9 expression in epidermal and dermal clusters by unbiased clustering.

C. Immunostaining shows MYH9 expression during skin organoid culture.

D. Immunostaining shows MYL9 and p-MYL9 expression during skin organoid culture.

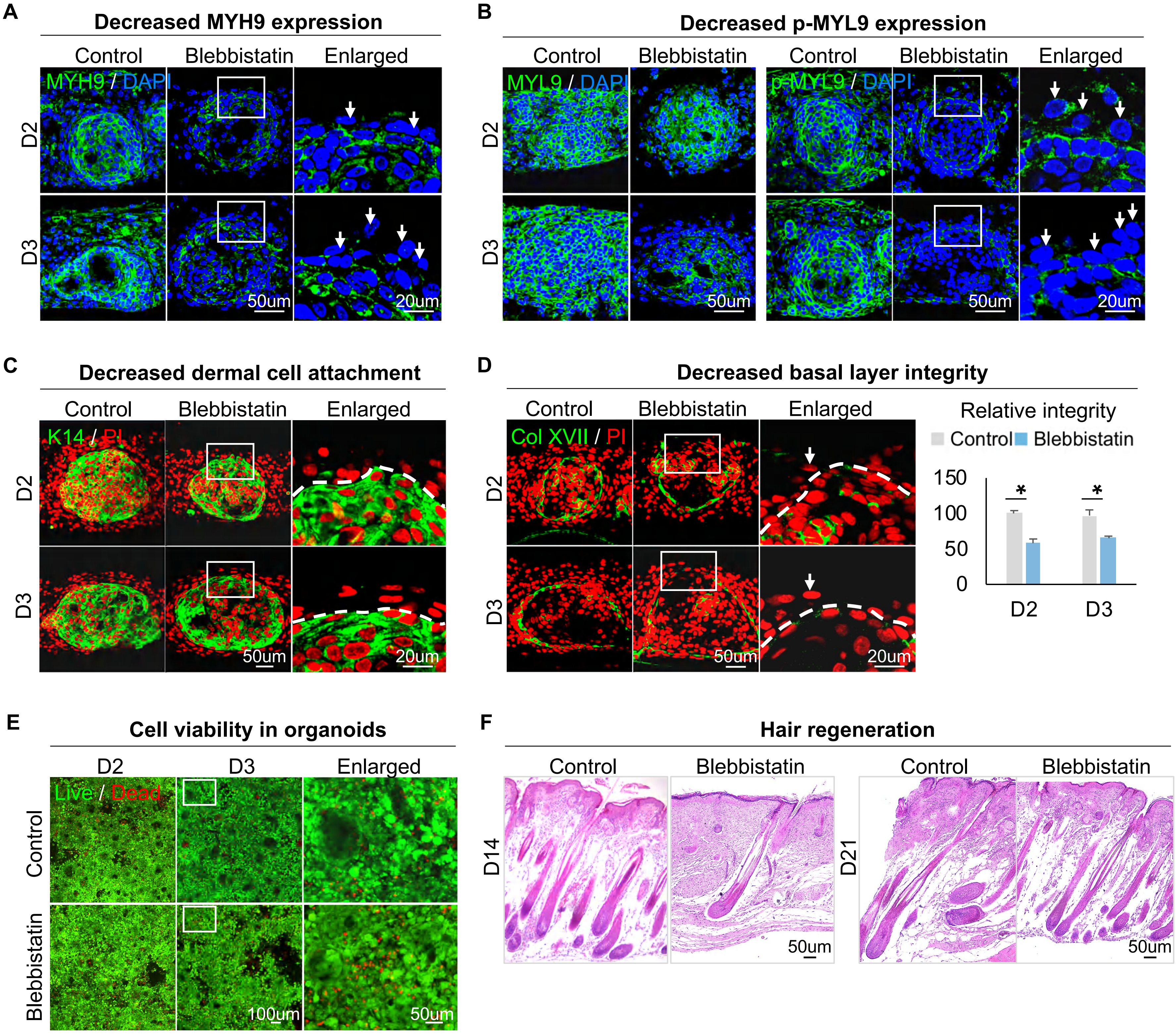

A
Decreased MYH9 expression
Control
Blebbistatin
Enlarged
MYH9 / DAPI
D2
D3
50um
20um
B
Decreased p-MYL9 expression
Control
Blebbistatin
Control
Blebbistatin
Enlarged
MYL9 / DAPI
p-MYL9 / DAPI
D2
D3
50um
50um
20um
C
Decreased dermal cell attachment
Control
Blebbistatin
Enlarged
K14 / PI
D2
D3
50um
20um
D
Decreased basal layer integrity
Control
Blebbistatin
Enlarged
Col XVII / PI
D2
D3
50um
20um
Relative integrity
Control
Blebbistatin
150
100
50
0
*
*
D2
D3
E
Cell viability in organoids
D2
D3
Enlarged
Live / Dead
Control
Blebbistatin
100um
50um
F
Hair regeneration
Control
Blebbistatin
Control
Blebbistatin
D14
D21
50um
50um

Figure S3. The decreased dermal cell attachment in skin organoid culture after treatment of Blebbistatin.

A. Immunostaining show MYH9 expression in Blebbistatin-treated cultures.

B. Immunostaining show MYL9 and p-MYL9 expression in Blebbistatin-treated cultures.

C. K14 immunostaining shows dermal cell attachment after treatment of Blebbistatin.

D. Collagen XVII immunostaining shows basal layer integrity after treatment of Blebbistatin (left). Statistics of the basal layer integrity (right). $N \geq 3$, $*p < 0.05$, and # no significant change.

E. Live-Dead staining shows cell survival after treatment of Blebbistatin.

F. H&E staining shows the hair cycle in the skin regenerated by the transplantation of Blebbistatin-treated cultures.

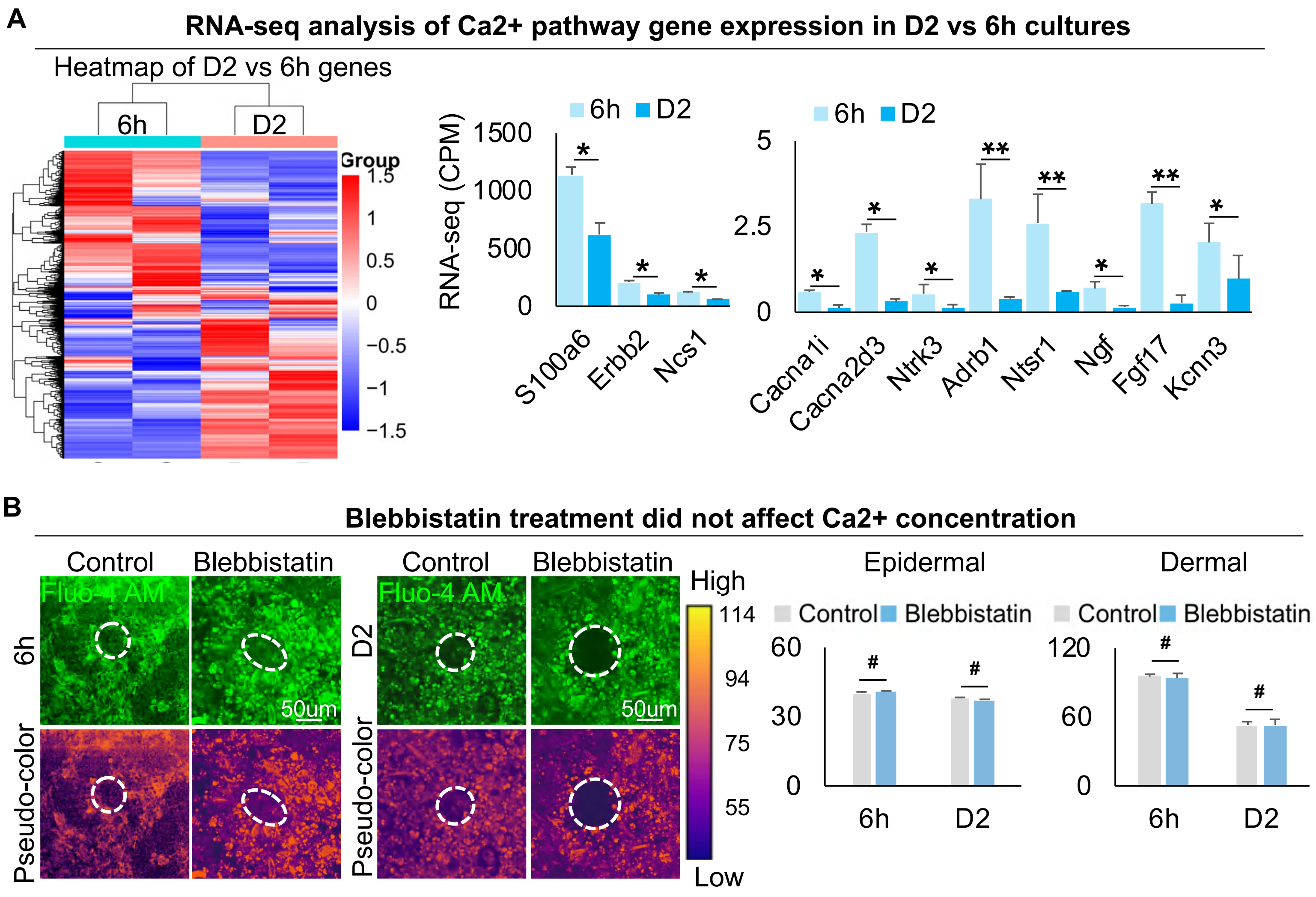

A
RNA-seq analysis of Ca2+ pathway gene expression in D2 vs 6h cultures
Heatmap of D2 vs 6h genes
6h
D2
Group
1.5
1
0.5
0
−0.5
−1
−1.5
RNA-seq (CPM)
6h
D2
1500
1000
500
0
S100a6
Erbb2
Ncs1
5
2.5
Cacna1i
Cacna2d3
Ntrk3
Adrb1
Ntsr1
Ngf
Fgf17
Kcnn3
B
Blebbistatin treatment did not affect Ca2+ concentration
Control
Blebbistatin
Fluo-4 AM
6h
D2
Pseudo-color
50um
High
114
94
75
55
Low
Epidermal
Dermal
Control
Blebbistatin
60
30
120
6h
D2
#

Figure S4. Bulk RNA-seq analysis shows Calcium pathway gene expression in 6h and D2 skin organoid culture.

A. Hierarchical clustering shows differential gene expression at D2 vs 6h (left). RNA-seq analysis of calcium pathway genes that are differentially expressed at D2 vs 6h (right).

B. Fluo-4 AM fluorescent probe detection assay and statistics show Ca^{2+} concentration within Blebbistatin cultures at 6h and D2. $N \geq 3$, # no significant change.

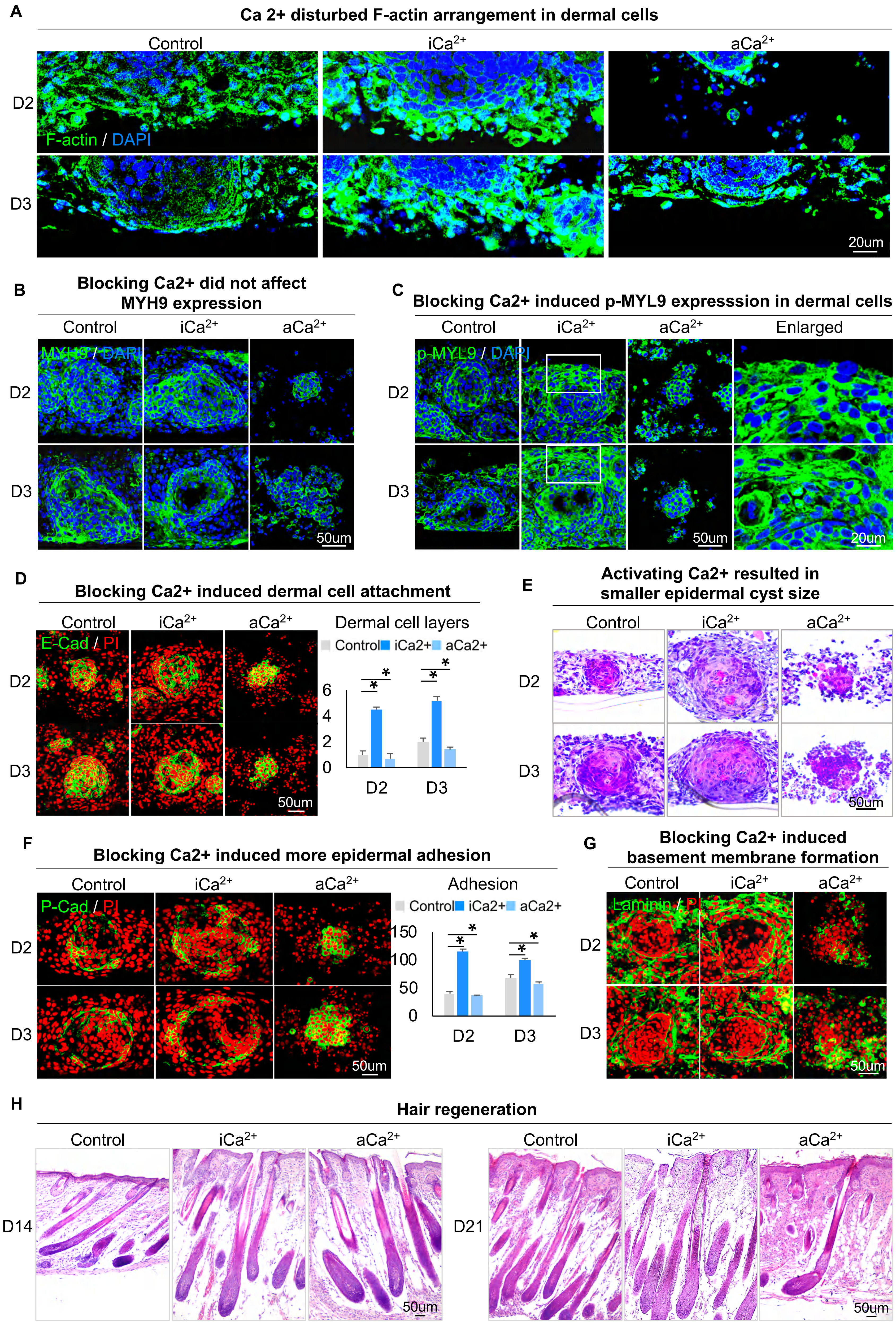
A
Ca 2+ disturbed F-actin arrangement in dermal cells
Control
iCa2+
aCa2+
D2
D3
F-actin / DAPI
20um
B
Blocking Ca2+ did not affect MYH9 expression
Control
iCa2+
aCa2+
D2
D3
MYH9 / DAPI
50um
C
Blocking Ca2+ induced p-MYL9 expresssion in dermal cells
Control
iCa2+
aCa2+
Enlarged
D2
D3
p-MYL9 / DAPI
50um
20um
D
Blocking Ca2+ induced dermal cell attachment
Control
iCa2+
aCa2+
E-Cad / PI
D2
D3
50um
Dermal cell layers
Control iCa2+ aCa2+
6
4
2
0
D2
D3
E
Activating Ca2+ resulted in smaller epidermal cyst size
Control
iCa2+
aCa2+
D2
D3
50um
F
Blocking Ca2+ induced more epidermal adhesion
Control
iCa2+
aCa2+
P-Cad / PI
D2
D3
50um
Adhesion
Control iCa2+ aCa2+
150
100
50
0
D2
D3
G
Blocking Ca2+ induced basement membrane formation
Control
iCa2+
aCa2+
Laminin / PI
D2
D3
50um
H
Hair regeneration
Control
iCa2+
aCa2+
D14
D21
50um

Figure S5. Calcium influences dermal cell attachment.

A. Enlarged views of immunostaining show F-actin arrangement after treatment with thapsigargin or ruthenium red.

B. Immunostaining shows MYH9 expression in thapsigargin- or ruthenium red-treated cultures.

C. Immunostaining shows MYL9 and p-MYL9 expression in thapsigargin- or ruthenium red-treated cultures.

D. E-Cadherin immunostaining shows more dermal cell attachment after blocking or activating the calcium signaling. Statistics of the dermal layers surrounding the epidermal cyst after treatment.

E. H&E staining shows a larger epidermal cyst in size after blocking calcium signaling.

F. P-Cadherin immunostaining shows higher multipotency after blocking or activating the calcium signaling. $N \geq 3$, $*p < 0.05$, and # no significant change.

G. Laminin immunostaining shows that blocking the calcium signaling promotes basement membrane formation.

H. H&E staining shows the hair cycle in the skin regenerated by the transplantation of the calcium signaling-blocked or activated cultures.

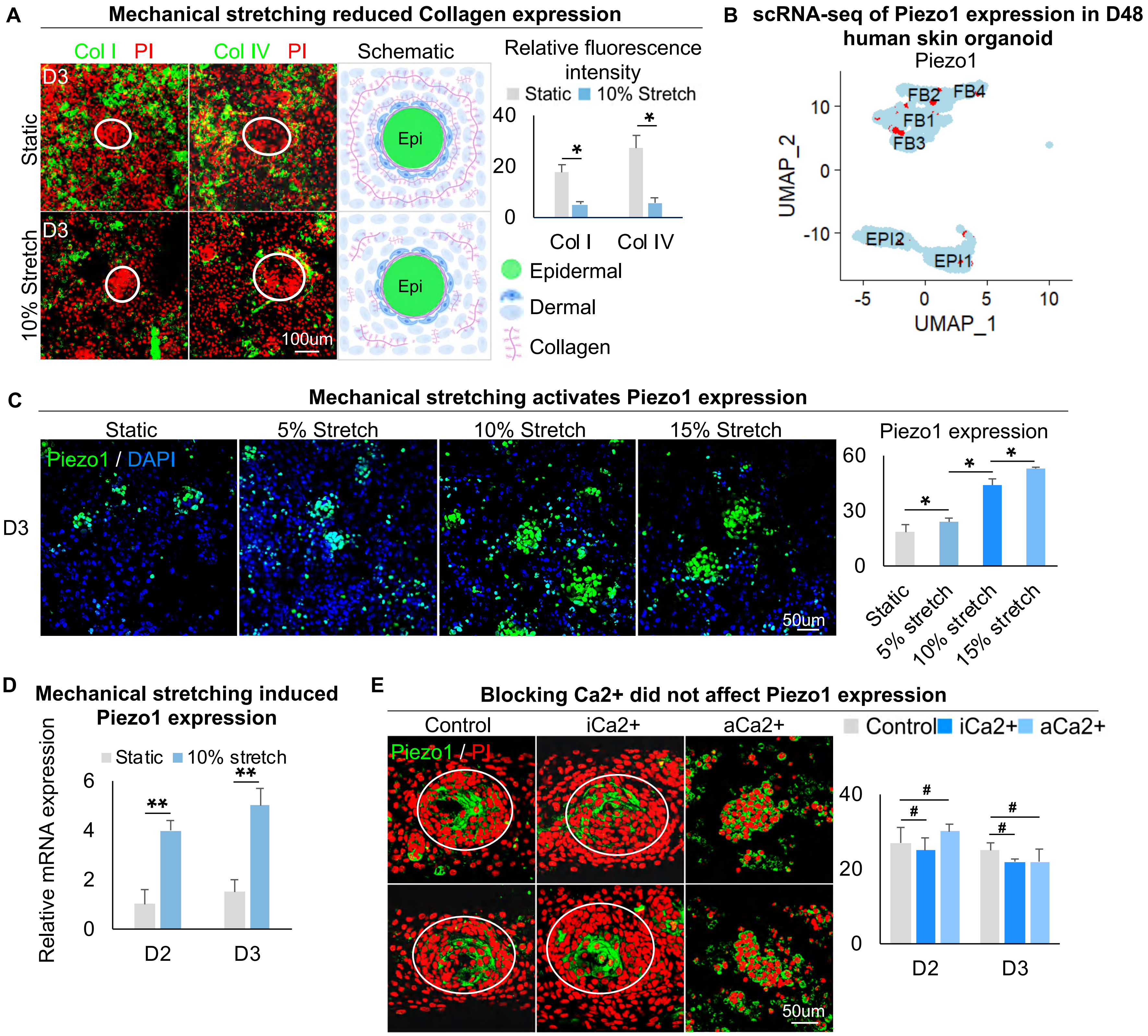
A
Mechanical stretching reduced Collagen expression
Col I PI
Col IV PI
Schematic
Relative fluorescence intensity
Static 10% Stretch
Static
10% Stretch
D3
Epi
100um
40
20
0
Col I
Col IV
Epidermal
Dermal
Collagen
B
scRNA-seq of Piezo1 expression in D48 human skin organoid
Piezo1
FB2
FB4
FB1
FB3
EPI2
EPI1
UMAP_2
UMAP_1
C
Mechanical stretching activates Piezo1 expression
Static
5% Stretch
10% Stretch
15% Stretch
Piezo1 / DAPI
D3
50um
Piezo1 expression
60
30
0
Static
5% stretch
10% stretch
15% stretch
D
Mechanical stretching induced Piezo1 expression
Static 10% stretch
Relative mRNA expression
6
4
2
0
D2
D3
E
Blocking Ca2+ did not affect Piezo1 expression
Control
iCa2+
aCa2+
Piezo1 / PI
50um
40
20
0
D2
D3

Figure S6. Mechanical stretching inhibits Collagen expression but induces Piezo1 expression.

A. Collagen I and Collagen IV immunofluorescence staining shows 10% mechanical stretching reduced Collagen expression. Schematic of Collagen expression after flexcell mechanical stretching.

B. UMAP plots of Piezo1 expression in epidermal and dermal clusters by unbiased clustering.

C. Immunostaining and statistics show that mechanical stretching induced Piezo1 expression under Static, 5%, 10%, or 15% Stretch.

D. Quantitative RT-PCR shows that the application of 10% mechanical stretching enhanced the expression of Piezo1 in skin organoid culture.

E. Immunostaining and statistical analysis show Piezo1 expression after blocking or activating the calcium signaling. $N \geq 3$, $**p < 0.01$, $*p < 0.05$, and # no significant change.

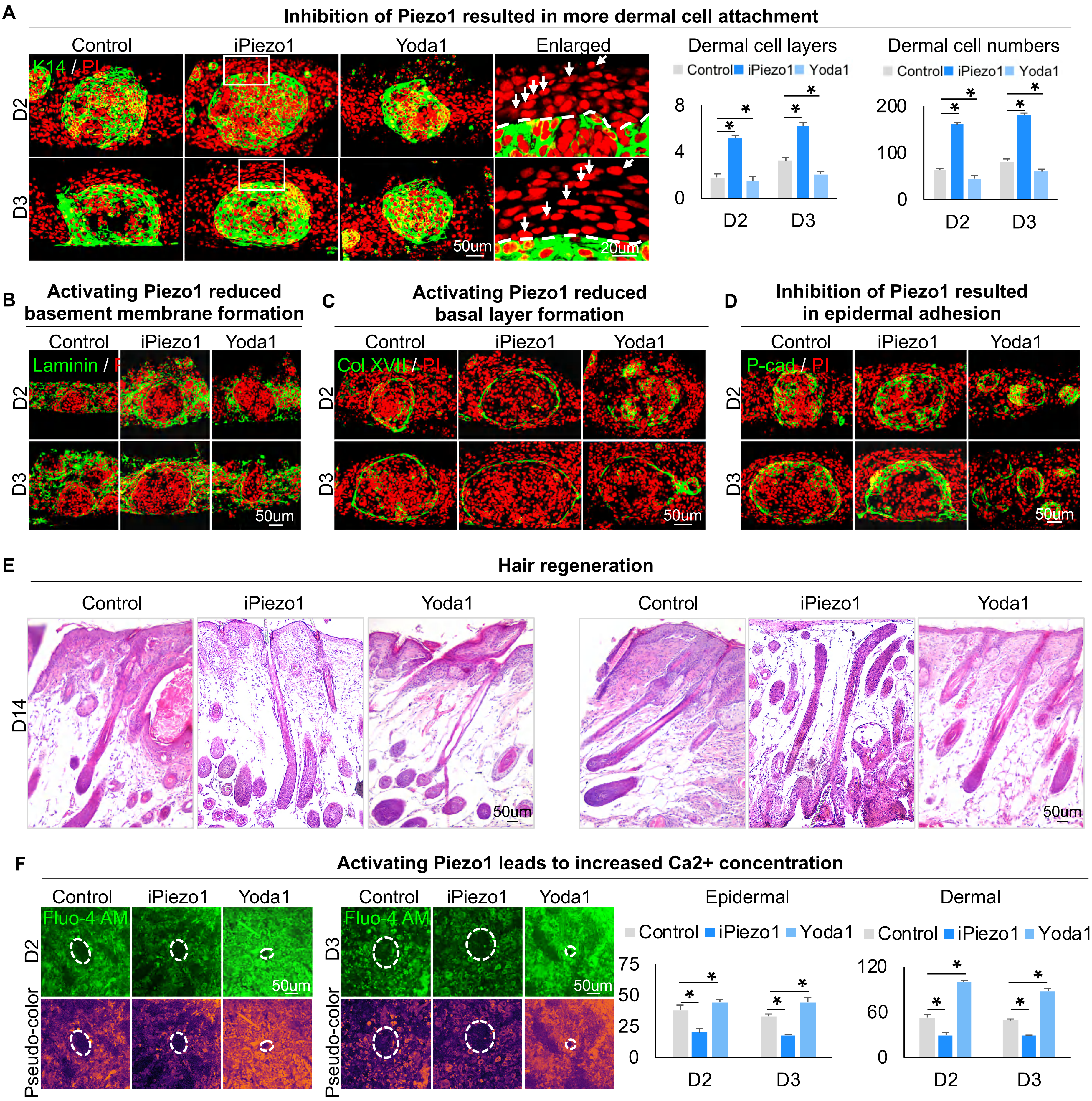

A
Inhibition of Piezo1 resulted in more dermal cell attachment
Control
iPiezo1
Yoda1
Enlarged
K14 / PI
D2
D3
50um
20um
Dermal cell layers
Control iPiezo1 Yoda1
8
4
0
D2
D3
Dermal cell numbers
Control iPiezo1 Yoda1
200
100
0
D2
D3
B
Activating Piezo1 reduced basement membrane formation
Control
iPiezo1
Yoda1
Laminin / PI
D2
D3
50um
C
Activating Piezo1 reduced basal layer formation
Control
iPiezo1
Yoda1
Col XVII / PI
D2
D3
50um
D
Inhibition of Piezo1 resulted in epidermal adhesion
Control
iPiezo1
Yoda1
P-cad / PI
D2
D3
50um
E
Hair regeneration
Control
iPiezo1
Yoda1
Control
iPiezo1
Yoda1
D14
50um
50um
F
Activating Piezo1 leads to increased Ca2+ concentration
Control
iPiezo1
Yoda1
Fluo-4 AM
D2
Pseudo-color
50um
Control
iPiezo1
Yoda1
Fluo-4 AM
D3
Pseudo-color
50um
Epidermal
Control iPiezo1 Yoda1
75
50
25
0
D2
D3
Dermal
Control iPiezo1 Yoda1
120
60
0
D2
D3

Figure S7. Piezo1 inhibits dermal cell attachment to the epidermal cysts.

A. K14 immunostaining shows more dermal cell attachment after blocking Piezo1. Statistics of the dermal layers and dermal cells surrounding the epidermal cyst after treatment.

B. Laminin immunostaining shows that blocking the Piezo1 promotes basement membrane formation.

C. Collagen XVII immunostaining shows that blocking the Piezo1 promotes basal layer formation.

D. P-Cadherin immunostaining shows higher multipotency after blocking Piezo1.

E. H&E staining shows the hair cycle in the skin regenerated by the transplantation of the Piezo1-blocked or activated cultures.

F. Ca^{2+} concentration was detected by the Fluo-4 AM fluorescent probe within Piezo1 inhibitor (GsMTx4 TFA) or Piezo1 activator (Yoda1) cultures at D2 and D3. $N \geq 3$, $*p < 0.05$, and # no significant change.

Table S1. Sequences of the Real-time qPCR.

**RT-qPCR Primers**		
**Gene Name**	**Forward**	**Reverse**
Piezo1	ACCAGTTTCTGGGACAAAACG	CCAGCCTGGTGGTGTTAAAGA
GAPDG	AGGTCGGTGTGAACGGATTTG	TGTAGACCATGTAGTTGAGGTCA

Table S2. Antibodies used in this study.

**Antibody**	**Isotype**	**Company**	**Cat #**
KRT14	Rabbit	Boster	A01432
Collagen I	Goat	Arigobio	ARG21965
Collagen IV	Rabbit	Abcam	ab19808
Collagen VII	Rabbit	Abcam	ab93350
Collagen XVII	Rabbit	Beyotime	AF1078
P-Cadherin	Goat	R&D	AF761
E-Cadherin	Mouse	Beyotime	AF0138
Laminin	Rabbit	Abcam	ab11575
Piezo1	Rabbit	Affinity	DF12083
MYL9	Rabbit	Proteintech	15354-1-AP
p-MYL9	Rabbit	Bioss	bs-4060R
MYH9	Rabbit	Beyotime	AF7536
F-actin	N/A	Invitrogen	A12379

Table S3. Small molecule inhibitors and activators used in this study.

**Cat #**	**Product Description**	**IC50**
SF9087	Blebbistatin (myosin II inhibitor)	IC50 = 0.5 µM-5 µM for inhibiting several striated muscle myosins as well as vertebrate nonmuscle myosin IIA and IIB.
HY-103311	Ruthenium Red (Calcium inhibitor)	IC50 = 7 µM for attenuating capsaicin-induced cation channel opening.
SC0389	Thapsigargin (Calcium activator)	IC50=30 nM for increasing in cytosolic calcium results from the specific inhibition of endoplasmic reticulum Ca^{2+}-ATPases.
S7399	GsMTx4 TFA (Piezo1 inhibitor)	GsMTx4 TFA (5 µM) reduces Piezo1-mediated charge transfer to 38% of its initial levels in HEK293 cells transfected with Piezo1 cDNA.
GC18233	Yoda1 (P1ezo1 activator)	EC50=17.1 µM, it is is a potent and selective Piezo1 agonist.
